# Oncolytic Maraba Virus MG1 Mediates Direct and Natural Killer Cell-Dependent Lysis of Ewing Sarcoma

**DOI:** 10.3390/cancers17203319

**Published:** 2025-10-14

**Authors:** Tyler Barr, Victoria A. Jennings, Elizabeth A. Roundhill, Richard T. Baugh, Maisa Yamrali, Heather E. Owston, Dennis McGonagle, Peter V. Giannoudis, Natasha J. Caplen, Javed Khan, John C. Bell, Susan A. Burchill, Fiona Errington-Mais, Graham P. Cook

**Affiliations:** 1 Leeds Institute of Medical Research, School of Medicine, University of Leeds, St James’s University Hospital, Leeds LS9 7TF, UKs.a.burchill@leeds.ac.uk (S.A.B.);; 2 Leeds Institute of Rheumatic and Musculoskeletal Medicine, School of Medicine, University of Leeds, St James’s University Hospital, Leeds LS9 7TF, UK; h.e.owston@leeds.ac.uk (H.E.O.);; 3 Genetics Branch, Center for Cancer Research, National Cancer Institute, NIH, Bethesda, MD 20814, USA; 4 Centre for Innovative Cancer Therapeutics, Ottawa Hospital Research Institute, Ottawa, ON K1H 8L6, Canada

**Keywords:** Ewing sarcoma, oncolytic virus, natural killer cells, tumour immunity, immunotherapy, Maraba virus

## Abstract

**Simple Summary:**

The five-year survival rates of Ewing sarcoma (EWS) patients, particularly those with metastatic and/or relapsed disease are poor and remain at less than 30%. This highlights a desperate need for new treatment approaches. The aim of this study was to investigate the efficacy of oncolytic Maraba virus strain, MG1, using in vitro models of EWS. Here, we demonstrate that MG1 exerts direct lytic effects on established EWS cell lines, doxorubicin resistant cell lines, spheroid cultures and primary patient-derived EWS cell cultures. We also explored the immune-stimulatory properties of MG1, where MG1 activated human healthy donor peripheral blood mononuclear cells and increased the immune-mediated destruction of EWS. These findings highlight the therapeutic potential of MG1 for EWS and warrant further investigation.

**Abstract:**

Background: Ewing sarcoma (EWS) is a rare cancer of the bone and soft tissue, most prevalent in children and young adults. The treatment of EWS has progressed relatively little in over 30 years. Survival rates for patients, particularly those with metastatic and/or relapsed disease remain poor, highlighting the urgent need for innovative treatment options. Methods: Here, we have explored the therapeutic potential of the oncolytic Maraba virus strain MG1 using various in vitro models of EWS, including established cell lines, doxorubicin-resistant derivatives, spheroid cultures and primary patient-derived Ewing sarcoma cell cultures. We examined the direct oncolytic activity of MG1 and its ability to stimulate the immune-mediated killing of EWS by human healthy donor peripheral blood mononuclear cells. Results: We show that MG1 undergoes productive replication and exerts direct oncolysis of established EWS cell lines, doxorubicin-resistant EWS cell lines and patient-derived Ewing sarcoma cell cultures more recently established from tumours. In contrast, primary mesenchymal stem cells (the likely cell of origin of EWS) were resistant to MG1, with IFN-I being a major determinant of tumour cell selectivity. MG1-treated PBMC produced IFN-I and killed EWS cells in vitro, in a natural killer (NK) cell-dependent manner. Conclusions: The ability of MG1 to kill EWS cells directly and stimulate NK cell cytotoxicity against this tumour suggests that MG1 may provide therapeutic benefit for EWS patients where the efficacy of conventional treatments is currently limited.

## 1. Introduction

Ewing sarcoma (EWS) was first described by James Ewing in 1921 [1]. This primary bone cancer predominantly affects children and young adults and is characterised by non-random chromosomal translocations between *FET* and *ETS* gene family members, resulting in the expression of fusion oncoproteins which drive many of the key features of EWS by acting as potent modulators of gene expression [2,3,4,5,6,7]. The treatment for EWS has changed very little in several decades, with combinations of chemotherapies (vincristine, ifosfamide, doxorubicin, etoposide and cyclophosphamide), radiotherapy and surgery being used [8,9]. Approximately 65–75% of patients with localised disease at diagnosis survive for five years or more [10]. However, patients with metastatic or relapsed disease have poor prognosis, with a 5-year survival rate of less than 30% [10]. This highlights a need for new treatment approaches, especially for patients with disseminated or relapsed/refractory disease.

Immune checkpoint inhibitors have revolutionised cancer immunotherapy and agents targeting programmed cell death protein-1 (PD-1), programmed death-ligand 1 (PD-L1) and cytotoxic T-lymphocyte associated protein-4 (CTLA-4) are approved for use in many solid tumour types [11]. Malignancies such as melanoma and non-small cell lung cancer, with high mutational burden and neoantigen load, generally respond well to immune checkpoint-based therapies.However, immune checkpoint inhibitors are less effective in EWS, most likely due to the low mutational burden and neoantigen load resulting in weak T cell responses [11,12,13,14,15].

Despite disappointing results for immune checkpoint-based therapies, there is mounting evidence of anti-tumour immune responses in EWS, supporting the idea that immunotherapeutic approaches may hold promise for patients. Cytotoxic lymphocytes (CD8+ T cells and natural killer [NK] cells) are present in the EWS tumour microenvironment (TME) and their presence is associated with better overall survival [16,17,18,19,20]. Moreover, rates of lymphocyte recovery post-chemotherapy treatment are associated with improved patient prognosis [21]. This indicates that EWS patients may indeed benefit from the application of immunotherapies, but different approaches may be required.

Oncolytic viruses (OVs) are a cancer immunotherapy which target tumours via two broad mechanisms, direct oncolysis associated with the release of progeny virions and the activation of anti-tumour immunity [22]. Moreover, OVs can reprogramme the TME by the induction of pro-inflammatory cytokines and chemokines, reducing immunosuppression and generating an immunologically “hot” TME [23]. The preference of OVs for cancer cells is not completely understood but is associated with the frequent inability of tumour cells to mount effective antiviral responses, thus making them permissive for viral replication [24,25,26]. By contrast, the OV-mediated production of type I interferon (IFN-I) by healthy cells stimulates innate immunity, in particular the activation of NK cells, which can detect and destroy both virally infected and malignant cells, placing them in a central position to mediate responses following OV infections of tumour cells [27,28].

The efficacy of OVs against EWS has been previously reported for a range of molecularly distinct OVs, including Maraba virus, protoparvovirus H-1, herpes simplex virus (rRp450), adenovirus (XVir-N-31), reovirus, vaccina virus, measles virus and vesicular stomatitis virus [29,30,31,32,33,34,35,36]. However, EWS cells are often included amongst a panel of other sarcoma types, and detailed studies focusing on OV and EWS have not been performed. Importantly, EWS cell lines can be recognised and killed by NK cells, but efficient killing requires NK cell activation by exogenous agents such as interleukin (IL)-2 or IL-15 [37,38,39]. We have previously demonstrated that OVs can activate human NK cells both in vitro and in vivo, suggesting that OVs might provide a useful stimulus to recruit NK cell activity against EWS [27,28,40].

Here, we have investigated the rhabdovirus, Maraba virus strain MG1, as a potential OV therapy for EWS in a range of human in vitro model systems. Maraba virus is a (-) single-stranded RNA (ssRNA) virus originally isolated from sandflies but, like other rhabdoviruses, has the capacity to replicate in mammalian cells [25]. Mutations in the virus M protein (L123W) and G protein (Q242R) were introduced to improve tumour cell selectivity, which is partially IFN-I dependent; this variant has the designation MG1 [25]. MG1 has direct oncolytic activity against many cancer cell lines derived from several tumour types [25,41,42]. Here, we describe the effects of MG1 on the direct and immune-mediated killing of EWS using established cell lines (cultured as both monolayers and spheroids), doxorubicin-resistant EWS cell lines and patient-derived Ewing sarcoma (PDES) cell cultures recently isolated from patient tumours. Our results demonstrate that MG1 exhibits efficient direct oncolysis and activates NK cell-dependent immune-mediated killing, suggesting that MG1 might provide a valuable therapeutic option for EWS.

## 2. Materials and Methods

Details of cell lines and primary cultures including growth medium are provided in Table S1. Details of other reagents including antibodies, fluorescent stains and buffers used in this study are detailed in Table S2.

### 2.1. Cell Culture

All cell cultures were routinely tested for mycoplasma using a Lookout^®^ One-Step Mycoplasma PCR Detection Kit (Sigma-Aldrich; Saint Louis, MO, USA) and were free from contamination. Unless otherwise stated, all growth mediums were supplemented with 10% foetal bovine serum (FBS; Sigma-Aldrich; Saint Louis, MO, USA), which was heat inactivated at 56 °C for 30 min before use. EWS cell lines SK-N-MC, TC-32, TTC-466 and SK-ES-1 were studied. All cell lines were short tandem repeat (STR) profiled to validate their authenticity using an American Type Culture Collection (ATCC) STR profiling service. Bone marrow-derived mesenchymal stem cells (MSCs) were isolated from human healthy donor bone marrow aspirates. Ethical approval was obtained from the NREC Yorkshire and Humberside National Research Ethics Committee (18/YH/0166). Isolation and analysis of MSCs was as previously described [43]. Peripheral blood mononuclear cells (PBMCs) were obtained from healthy donor leukocyte apheresis cones supplied by the National Health Service Blood and Transplant unit (NHSBT). PBMCs were isolated using Lymphoprep^TM^ (STEMCELL Technologies; Cambridge, UK) and density gradient centrifugation. Patient-derived EWS (PDES) cell cultures were isolated from treatment-naïve EWS patient biopsy tissue, as previously described [44], validation of EWS phenotypes including fusion status and CD99 expression have been previously described [45]. Informed consent and ethical approval for the collection of the tumours and generation of PDES cell cultures was obtained through GenoEWING (IRAS 167880, EDGE 79301). Doxorubicin-resistant cell lines were generated by maintaining SK-N-MC and TC-32 cell lines in half maximal effective concentrations (EC50) of doxorubicin as determined by trypan blue exclusion for up to 6 months, and were then re-challenged with the 10-fold EC50 of doxorubicin until the EC50 population was stable, as previously described [44]. Three-dimensional (3D) spheroid cultures were generated by seeding 2 × 10^3^ EWS cells into a 96 well U bottom ultra-low adhesion plate (Corning Incorporated; Corning, NY, USA) for 7 days. Half media changes were carried out every 2–3 days.

### 2.2. Oncolytic Maraba Virus

MG1-GFP and MG1-firefly luciferase (MG1-FLUC) were amplified on Vero cells. A standard plaque assay on the Vero cell line was used to assess the viral titre of MG1 stocks and MG1-treated cell lysates. The virus backbone has previously been described [25].

### 2.3. Assessment of Cell Viability and Growth Inhibition

Cell viability was evaluated using a LIVE/DEAD^®^ Fixable Yellow Dead Cell Stain Kit (Thermo Fisher Scientific Inc; Waltham, MA, USA). Growth inhibition of monolayer cultures was measured using Methylthialazole Tetrazolium (MTT; Sigma-Aldrich; Saint Louis, MO, USA) assay and CellTiter-Glo^®^ reagent (Promega; Madison, WI, USA) for spheroid cultures. All assays were conducted as per the manufacturers’ instructions.

### 2.4. NK Cell Depletion and Flow Cytometry-Based Functional Assays

NK cells were depleted from whole PBMCs using anti-CD56 magnetic beads (Miltenyi Biotec; Bergisch Gladbach, Germany). Briefly, PBMCs were labelled with anti-CD56 beads in MACS buffer for 15 min. Cells were then passed through LS columns (Miltenyi Biotec; Bergisch Gladbach, Germany), mounted on a magnet and washed 3 times with MACS buffer. Flow through was collected, and CD56+ NK cell depletion was validated using anti-CD3 and anti-CD56 antibodies and flow cytometry. For immune-killing assays, whole or NK cell-depleted PBMCs were treated with MG1 for 48 h. PBMCs were then co-cultured with CellTracker™ Green CMFDA Dye (Thermo Fisher Scientific Inc; Waltham, MA, USA)-stained EWS targets at a ratio of 25:1 for 5 h. PBMC-EWS co cultures were then stained with a LIVE/DEAD™ Fixable Yellow Dead Cell Stain Kit (Thermo Fisher Scientific Inc; Waltham, MA, USA) for 30 min at 4 °C then washed in PBS and fixed in 1% paraformaldehyde (PFA).

For NK cell degranulation assays, PBMCs were co-cultured with targets at a ratio of 10:1 for 1 h, before the addition of anti-CD3, anti-CD56 and anti-CD107a antibodies and Brefeldin A at a final concentration of 3 µg/mL (Table S2). Co-cultures were incubated for a further 3 h, and cells were then washed in PBS and fixed in 1% PFA.

For the assessment of NK cell activation, PBMCs were treated with MG1 at 0, 0.01, 0.1 and 1 PFU/cell MG1 for 48 h. Cells were then stained with anti-CD3, anti-CD56, ant-CD69 and anti-CD317 antibodies (Table S2) for 30 min at 4 °C, then washed in PBS and fixed in 1% PFA.

For all flow cytometry-based assays, cells were analysed using a Cytoflex LX (Beckman Coulter; Brea, CA, USA) or Attune (Thermo Fisher Scientific Inc; Waltham, MA, USA) flow cytometers and analysed using FlowJo V10.8.1 and CytExpert V2.5 software.

### 2.5. ELISA

IFNα ELISA was carried out using Maxisorp plates and matched paired antibodies (Table S2). Detection of IFNβ in cell supernatants was carried out using a Human IFNβ ELISA Kit (R&D Sytems; Minneapolis, MN, USA).

### 2.6. Transcriptome Analysis

Expression of the CD99 and LDLR genes was analysed using bulk RNA sequencing (RNAseq) data from tumours and cell lines [46,47] and single-cell RNAseq (scRNAseq) data from tumours [19]. The Coefficient of Variation (CV) of expression was calculated from the standard deviation divided by the mean of expression for each gene.

### 2.7. Statistical Analysis

Significant differences in results were determined using a Student’s *t*-test, one-way ANOVA or two-way ANOVA with Tukey’s post hoc test. Correlations were determined using a Pearson’s correlation coefficient (r). IC50 values were calculated using regression analysis, applying a best fit line. Statistical analyses were performed using GraphPad PRISM 10 software.

## 3. Results

### 3.1. EWS Cell Lines Are Sensitive to MG1 Infection, Replication and Oncolysis

**Figure 1:**
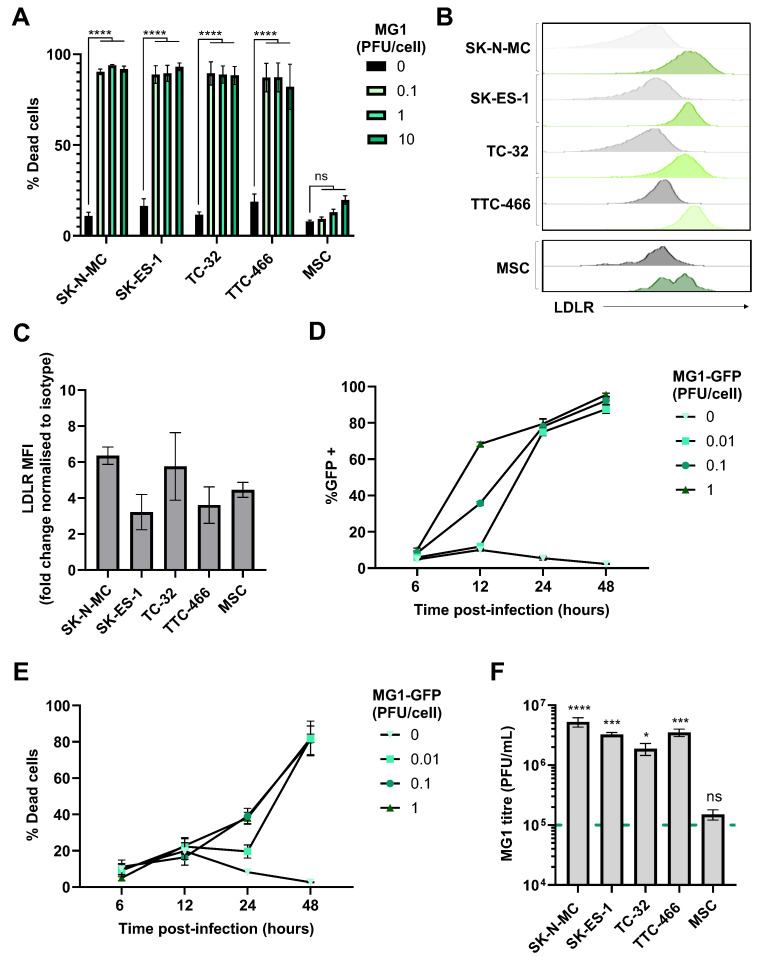
EWS cell lines are susceptible to the direct oncolytic effects of MG1. (**A**) Four EWS cell lines (SK-N-MC, SK-ES-1, TC-32 and TTC-466) and primary healthy donor MSCs were treated ± MG1 at 0.1, 1 or 10 plaque forming unit (PFU)/cell. After 48 h, cells were stained with the LIVE/DEAD™ Fixable Yellow Dead Cell Stain and analysed using flow cytometry, the graph shows mean ± standard error of the mean (SEM), *n* = 3, a two-way ANOVA was performed **** *p* < 0.0001; ns, not significant. (**B**,**C**) EWS cell line and MSC expression of MG1 viral entry receptor LDLR was assessed using fluorescently conjugated antibodies and a matched isotype control, expression was quantified using flow cytometry, (**B**) grey = isotype control, green = LDLR. (**C**) Summary of expression of LDLR flow cytometry; the y axis shows the fold change in mean fluorescence intensity (MFI) of LDLR expression compared to staining using the isotype control antibody, results show mean ± SEM, *n* = 3. (**D**,**E**) TC-32 cell lines were treated ± MG1-GFP at 0.01, 0.1, 1 PFU/cell. Cells were harvested at 6, 12, 24 and 48 h post infection and stained with the LIVE/DEAD™ Fixable Yellow Dead Cell Stain. Shown are the percentage of (**D**) GFP positive MG1-infected cells and (**E**) % dead cells, which was quantified using flow cytometry, results show mean ± SEM, *n* = 3. (**F**) EWS cell lines and MSC were treated with 1 PFU/cell MG1 for 48 h, cell supernatants were collected and MG1 titre assessed using standard plaque assay on the Vero cell line. Dashed green line represents MOI of viral input. The graph shows mean ± SEM, *n* = 3, a one-way ANOVA was performed to compare viral titre to viral input, * *p* < 0.05, *** *p* < 0.001 and **** *p* < 0.0001; ns, not significant.

Four, long-established EWS cell lines were tested for their susceptibility to direct oncolysis by MG1. SK-N-MC, SK-ES-1 and TC-32 cell lines harbour the t(11;22)(q24;q12) EWSR1::FLI1 oncogenic fusion event, and TTC-466 the t(21;22)(q22;q12) EWSR1::ERG fusion oncogene. Sarcomas are derived from mesenchymal tissues and the EWS cell of origin has been reported to be mesenchymal stem cells (MSCs; also known as mesenchymal stromal cells) [3,48]. We therefore compared the MG1-mediated lysis of bone marrow-derived MSCs from a healthy donor, with that of EWS cell lines to validate the tumour-selective oncolytic effects of MG1. EWS cell lines and MSCs were infected with MG1 at an increasing multiplicity of infection (MOI) and cell death was analysed 48 h post-infection by flow cytometry. All four EWS cell lines were susceptible to MG1 infection and oncolysis, even at the lowest titre of 0.1 plaque forming units (PFUs) per cell (Figure 1A; *p* < 0.0001). By contrast, the MSCs were resistant to MG1 oncolysis, consistent with the tumour-selective activity of MG1 (Figure 1A). Importantly, MG1 exhibited significantly increased levels of oncolysis when compared with a range of other molecularly distinct OVs, including reovirus, HSV1716 and Coxsackievirus-A21, justifying the prioritisation of this OV in this study (Figure S1A).

MG1 entry into cancer cells requires the ubiquitously expressed low-density lipoprotein receptor (LDLR), and all the long-established cell lines and MSCs expressed cell surface LDLRs as determined by flow cytometry (Figure 1B,C). We analysed the kinetics of the MG1 infection of EWS using a GFP-encoding MG1 (MG1-GFP). Viral gene expression was detectable ~12 h post infection and cell death occurred ~24 h post infection (Figure 1D,E and Figure S1B). Supernatants from MG1-infected cells were screened for MG1 output 48 h post-infection, demonstrating a significant increase in viral titre for all EWS cell lines, with a greater than 10-fold increase in virus relative to the input (*p* < 0.05; Figure 1F). Furthermore, replication was tumour-selective, with no significant increase in viral titre observed following the MG1 treatment of MSCs, despite their expression of LDLRs, indicating that while the expression of this receptor may be necessary for viral infection, other cellular factors modulated in EWS cells may mediate sensitivity to replication and oncolysis (Figure 1C,F). While the assessment of MG1 oncolysis in MSCs is limited to those derived from a single donor, the heterogeneity of MSC cultures from individual bone marrow donors is well established and is confirmed by the observed heterogenous expression of LDLRs (Figure 1B) [49].

### 3.2. MG1 Retains Oncolytic Effects Against Chemotherapy-Resistant and Spheroid-Cultured EWS Cell Lines

**Figure 2:**
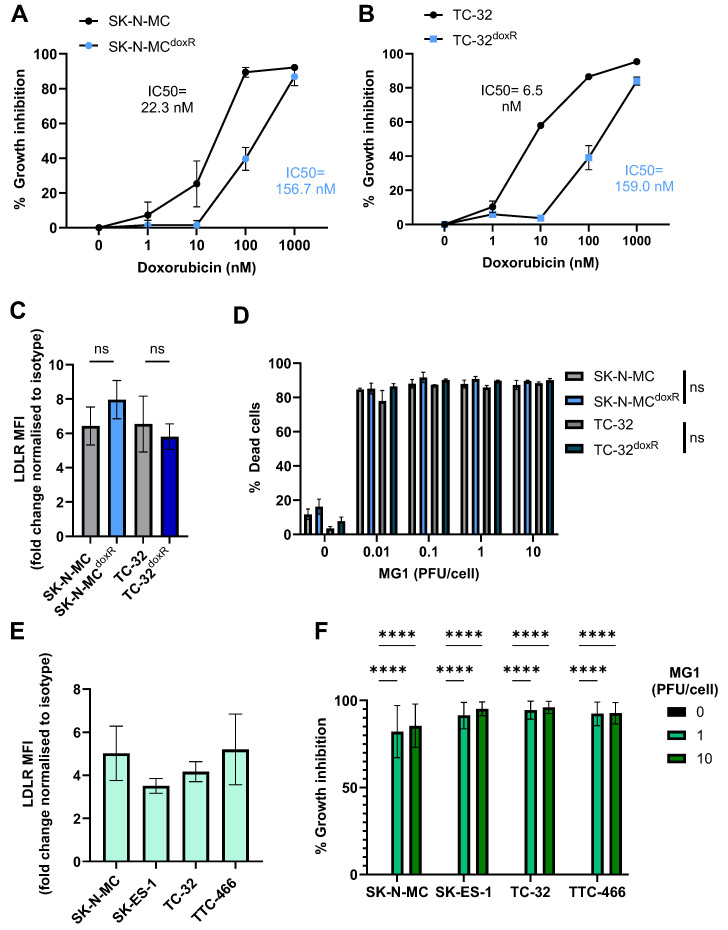
Doxorubicin-resistant EWS cell lines and spheroid cultures retain sensitivity to MG1 oncolysis. (**A**,**B**) Doxorubicin-resistant (SK-N-MC^doxR^ and TC-32^doxR^) and parental (SK-N-MC and TC-32) cell lines were treated with doxorubicin concentrations ranging from 0 to 1000 nM for 48 h. Growth inhibition was assessed using an MTT assay, results presented as mean ± SEM, *n* = 3, IC50 values calculated using nonlinear regression analysis. (**C**) Expression of MG1 entry receptor LDLR on doxorubicin-resistant and parental cell lines was assessed using flow cytometry, results show the fold change in MFI relative to isotype controls. Presented as mean ± SEM, *n* = 3, paired *t*-tests performed. (**D**) Doxorubicin-resistant and parental cell lines were treated ± MG1 at 0.01, 0.1, 1 and 10 PFU/cell for 48 h. Cells were stained with the LIVE/DEAD™ Fixable Yellow Dead Cell Stain and cell death was determined by using flow cytometry, presented as mean ± SEM, *n* = 3, multiple paired *t*-tests performed. (**E**) Expression of LDLR on EWS spheroid cultures was assessed by dissociation of spheroids using Accutase. Dissociated cells were stained with the LIVE/DEAD™ Fixable Yellow Dead Cell Stain and fluorescently conjugated anti-LDLR antibodies, and a matched isotype control, results show the fold change in MFI on live cells, relative to isotype controls, presented as mean ± SEM, *n* = 3. (**F**) EWS spheroids were treated ± MG1 at 1 or 10 PFU/cell for 48 h and CellTiter-Glo^®^ reagent was used to measure growth inhibition. Growth inhibition calculated as percentage luminescence relative to untreated control. Presented as mean ± SEM, *n* = 3, a two-way ANOVA was performed **** *p* < 0.0001.

As with many other cancers, acquired resistance to chemotherapy presents a major challenge to successful EWS treatment [50]. Therefore, we generated SK-N-MC and TC-32 cell lines with resistance to doxorubicin, producing cell lines that were ~7 times (for SK-N-MC^doxR^) and ~24 times (for TC-32^doxR^) more resistant to doxorubicin chemotherapy than their parental counterparts, based on IC_50_ values (Figure 2A,B). Importantly, these doxorubicin resistant derivatives did not display any significant differences in cell surface LDLR expression or sensitivity to MG1 oncolysis (Figure 2C,D).

Spheroid cultures more closely mimic solid tumour architecture than monolayer cultures, and demonstrate greater resistance to certain therapeutics, for example, showing reduced susceptibility to chemotherapy [51]. Moreover, MG1 cytotoxicity has been reported to be reduced in ovarian cancer spheroids, as a result of reduced LDLR expression; therefore, we wanted to investigate if similar effects were seen in EWS [52]. We generated spheroids from the four long-established cell lines (Figure S2). Unlike results from ovarian cancer, the EWS cell spheroids expressed LDLRs and remained susceptible to MG1 oncolysis (Figure 2E,F). These results demonstrate that MG1 infects and kills EWS in both conventional 2D culture conditions and when grown as 3D spheroids.

### 3.3. PDES Cell Cultures Express LDLR and Retain Sensitivity to MG1 Oncolysis

**Figure 3:**
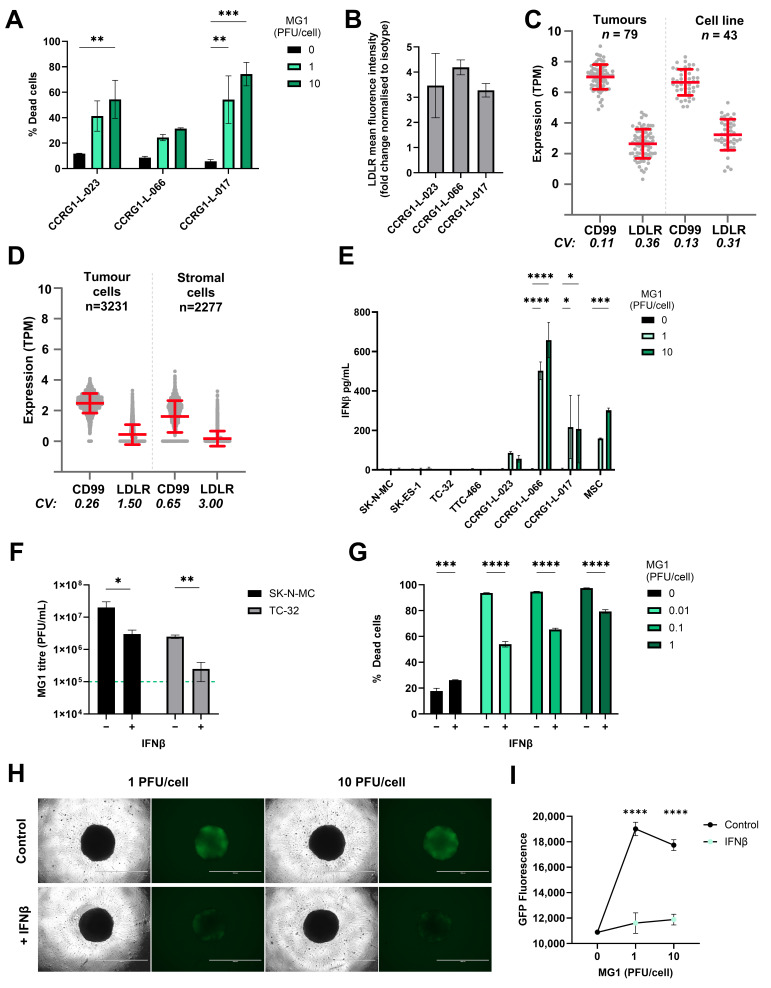
Sensitivity of EWS to MG1 oncolysis is modulated by IFNβ. (**A**) PDES cell cultures were treated ± MG1 at 1 or 10 PFU/cell for 48 h. Cells were stained with the LIVE/DEAD™ Fixable Yellow Dead Cell Stain and cell death was assessed using flow cytometry. Presented as mean ± SEM, *n* = 3, a two-way ANOVA was performed ** *p* < 0.01, *** *p* < 0.001. (**B**) PDES cell culture expression of viral entry receptor LDLRs assessed using flow cytometry, results show the fold change in MFI relative to isotype controls. Presented as mean ± SEM, *n* = 3 (**C**,**D**) Expression of CD99 and LDLR genes was analysed using RNA sequencing data, (**C**) bulk RNAseq data from 43 EWS cell lines and 79 EWS tumour samples and (**D**) single-cell RNA sequencing data from 3231 tumour cells and 2277 stromal cells. The Coefficient of Variation (CV) of expression was calculated from the standard deviation divided by the mean of expression for each gene. (**E**) PDES cell cultures and established cell lines were treated ± MG1 at 1 or 10 PFU/cell and cell-free supernatants were collected after 48 h and screened for IFNβ using ELISA. Presented as mean ± SEM, *n* = 3, a two-way ANOVA was performed * *p* < 0.05, *** *p* < 0.001 and **** *p* < 0.0001. (**F**) TC-32 and SK-N-MC cells were treated ± IFNβ at 400 pg/mL for 24 h and then treated ± MG1 at 1 PFU/cell for 24 h. Cell-free supernatants were screened for MG1 using a plaque assay to assess viral replication, the green dashed line represents titre of viral input. Presented as mean ± SEM, *n* = 3, paired *t*-tests were performed * *p* < 0.05, ** *p* < 0.01. (**G**) TC-32 cells were treated ± IFNβ at 400 pg/mL for 24 h, and then treated ± MG1 at 0.01, 0.1 and 1 PFU/cell for 48 h. Cells were stained with LIVE/DEAD™ Fixable Yellow Dead Cell Stain and analysed using flow cytometry. Presented as mean ± SEM, *n* = 3, a two-way ANOVA was performed *** *p* < 0.001, **** *p* < 0.0001. (**H**,**I**) TC-32 cells were seeded into low adhesion 96 well plates at 2000 cells/well to generate spheroids over 7 days. Spheroids were treated ± IFNβ at 400 pg/mL for 24 h and then treated ± MG1-GFP at 1 or 10 PFU/cell. After 24 h, (**H**) cells were imaged to assess GFP fluorescence using an EVOS microscope at 4x magnification, the scale bar represents 1 mm. (**I**) GFP fluorescence was quantified using a Cytation 5 plate reader. Presented as mean ± SEM, *n* = 3, a two-way ANOVA was performed **** *p* < 0.0001.

Next, we investigated the ability of MG1 to infect and kill PDES cell cultures. PDES cultures were more recently established from patient samples and for these studies we used three PDES cell cultures, two of which express EWSR1::FLI1 fusion oncoprotein (CCRG1-L-017 and CCRG-L-023) and one which expresses EWSR1::ERG fusion oncoprotein (CCRG1-L-066) [45]. All of the PDES cell cultures express cell surface CD99 (a protein over-expressed in EWS tumours and used in diagnosis), albeit with greater heterogeneity than the established cell lines (Figure S3A). The PDES cells are transcriptionally distinct from long-established EWS cell lines and are more resistant to chemotherapy [45,53]. Furthermore, PDES cell cultures have a different forward and side scatter profile when assessed by flow cytometry and microscopy demonstrating that they are larger with a more elongated, mesenchymal-like morphology (Figure S3B,C). Importantly, all three PDES cell cultures demonstrated susceptibility to MG1, which was consistent with LDLR expression (Figure 3A,B).

To determine the potential utility of MG1 as a therapeutic agent for EWS patients, we next evaluated LDLR and CD99 expression in a larger cohort of EWS samples using transcriptome data. CD99 is highly expressed in EWS cells, where it serves as a key diagnostic marker. However, its expression is not limited to EWS; it is also found on a variety of other cell types including immune cells, mesenchymal cells and endothelial cells [54]. A cohort of 79 EWS tumours and 43 EWS cell lines [47] showed that CD99 and LDLR gene expression was detected in all of the patient samples and established cell lines tested (Figure 3C). Furthermore, to address heterogeneity at the single-cell level we analysed EWS scRNAseq data [19]. Within this dataset, Visser et al. identified both tumour cells (*n* = 3231) and stromal cells (*n* = 2277), the latter comprising mesenchymal cells, endothelial cells and multiple immune cell types. The expression of both CD99 and LDLR genes was variable in the stroma, reflecting the multiple cell types present in this compartment (Figure 3D). As expected, CD99 expression was relatively uniform in the EWS tumour cells (98% of tumour cells expressed CD99), but only 38% of EWS tumour cells expressed detectable levels of LDLR mRNA (Figure 3D). Heterogeneity of LDLR gene expression at the single-cell level suggests that not all malignant cells within the EWS tumour will be directly targetable by MG1 infection and oncolysis.

### 3.4. EWS Sensitivity to Maraba Virus Is Counteracted by IFNβ Responses

The long-established EWS cell lines and PDES cultures expressed LDLR but displayed variable susceptibility to MG1. For example, treatment with 1 PFU/cell MG1 was sufficient to kill ~80–90% of the population of the established cell lines (Figure 1A), whereas the same titre killed less than 60% of the population in PDES cell cultures (Figure 3A). We speculated, given the known IFN-I sensitive nature of MG1 previously reported, that the PDES cell cultures might retain some anti-viral activity and thereby restrict MG1 replication [25]. In support of this, all three PDES cultures (and the MSC culture) produced detectable levels of IFNβ in response to MG1 infection, whereas no IFNβ was detected upon infection of the established cell lines (Figure 3E). None of the cell types tested secreted IFNα in response to MG1 (Figure S4A).

The importance of IFNβ in determining the susceptibility to MG1 was further investigated by performing infections in the presence or absence of exogenous IFNβ; TC-32 and SK-N-MC cell lines were pre-treated with recombinant human IFNβ for 24 h before MG1 infection and tested for the effect of this IFN-I on viral replication and oncolysis. These data show that IFNβ treatment significantly reduced MG1 replication and oncolysis in established EWS cell lines (Figure 3F,G and Figure S4B). This effect was also observed in spheroids, where IFNβ pre-treatment of TC-32 and SK-N-MC spheroid cultures significantly reduced MG1-GFP replication (Figure 3H,I and Figure S4C,D). These results demonstrate that the anti-viral capacity of the EWS tumour cells is an important determinant of their susceptibility to MG1.

### 3.5. MG1 Activates NK Cells and Granule Mediated Destruction of EWS

**Figure 4:**
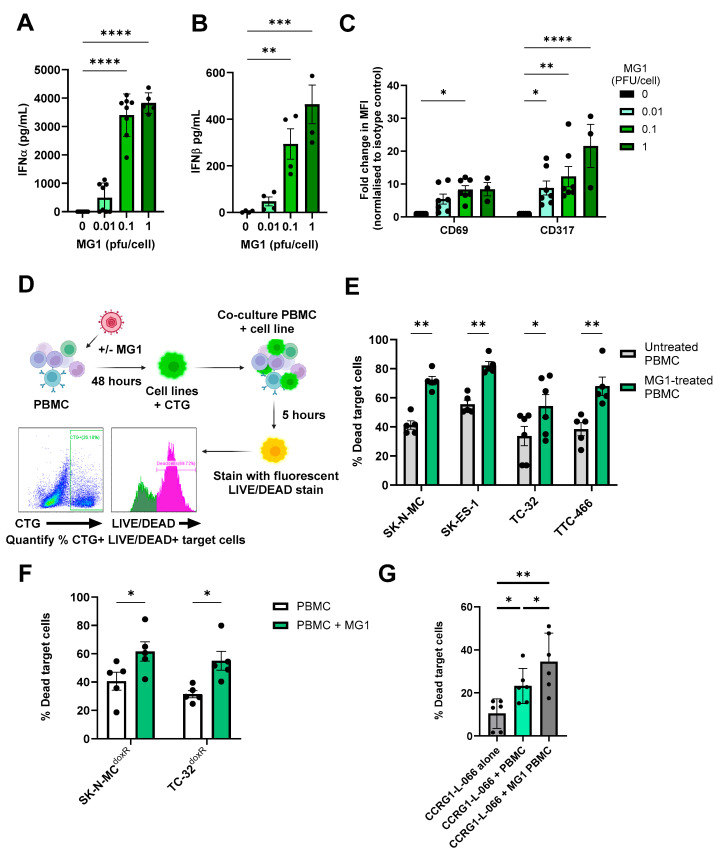
MG1 treatment stimulates the immune-mediated killing of EWS. (**A**–**C**) PBMCs were treated ± MG1 at 0.01, 0.1 or 1 PFU/cell for 48 h. (**A**,**B**) Cell-free supernatants were collected and (**A**) IFNα and (**B**) IFNβ detected using ELISA. Presented as mean ± SEM, *n* ≥ 3, a one-way ANOVA was performed, ** *p* < 0.01, *** *p* < 0.001 and **** *p* < 0.0001. (**C**) Cells were stained with CD56, CD3, CD69 or CD317 antibodies, or matched isotype controls. Cells were analysed to assess MFI of CD69 and CD317 activation markers on NK cells (CD56+CD3-) using flow cytometry. Presented as mean ± SEM, *n* ≥ 3, a two-way ANOVA was performed, * *p* < 0.05, ** *p* < 0.01 and **** *p* < 0.0001. (**D**) Schematic showing process of immune killing assays. Briefly, PBMCs were treated ± 1 PFU/cell MG1 for 48 h and then co-cultured with cell tracker green (CTG)-labelled EWS cells for 5 h. Cells were then stained with the LIVE/DEAD™ Fixable Yellow Dead Cell Stain, and the percentage of dead target cells was quantified by flow cytometry. Created in https://BioRender.com. Immune killing assay of (**E**) EWS cell lines. Presented as mean ± SEM, *n* ≥ 5, a two-way ANOVA was performed, * *p* < 0.05, ** *p* < 0.01. (**F**) doxorubicin-resistant cell lines. Presented as mean ± SEM, *n* = 5, a two-way ANOVA was performed, * *p* < 0.05 and (**G**) patient-derived cell culture CCRG-L-066. Presented as mean ± SEM, *n* = 6, a paired test was performed * *p* < 0.05, ** *p* < 0.01. All results show the mean ± SEM for a minimum of *n* = 3 independent experiments.

Although IFN-I can limit viral replication, its production is critical for triggering host anti-tumour immune responses, including NK cell activation and cytotoxicity [27,55]. To test this possibility in EWS, we treated PBMCs isolated from healthy donors with an increasing MOI of MG1 for 48 h. As expected, this resulted in production of IFN-I (IFNα and IFNβ) and expression of the IFN-I inducible molecules CD69 and CD317/tetherin on the NK cell surface (Figure 4A–C). To examine NK cell cytotoxicity, we repeated treatment of PBMCs with MG1 (at 1 PFU/cell) for 48 h and performed an immune cytotoxicity assay by co-culturing the virus-stimulated PBMCs with EWS target cells for 5 h and assaying EWS cell death (Figure 4D). Importantly, MG1 does not kill EWS cell lines within 5 h (as shown in Figure 1A) and hence, this assay reflects PBMC-mediated killing and not virus-mediated oncolysis; PBMC-mediated killing of SK-N-MC, SK-ES-1, TC-32 and TTC-466 cells was significantly enhanced following the pre-treatment of PBMCs with MG1 (Figure 4E). Furthermore, doxorubicin-resistant cell cultures SK-N-MC^doxR^ and TC-32^doxR^ and PDES cell cultures were also sensitive to the action of MG1 treated PBMCs (Figure 4F,G).

**Figure 5:**
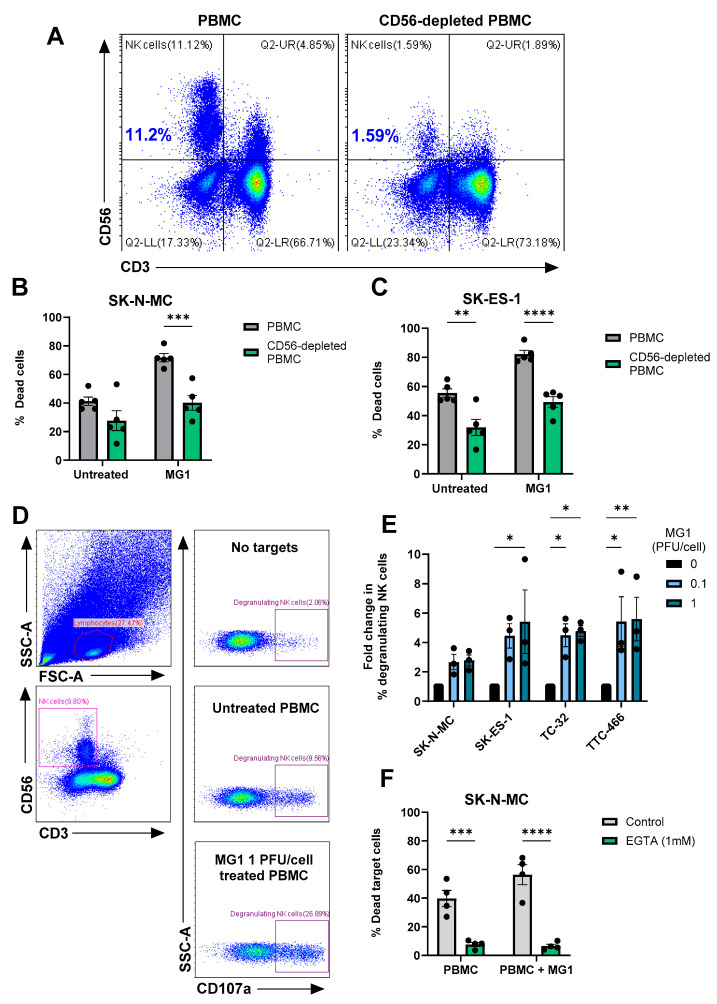
MG1 stimulates immune-mediated killing of EWS targets by NK cell degranulation. (**A**–**C**) NK cells were depleted from PBMCs using CD56 magnetic bead selection and PBMCs were treated ± MG1 at 1 PFU/cell for 48 h. (**A**) Depletion was validated using anti-CD56 and anti-CD3 antibodies and flow cytometry. (**B**,**C**) Whole PBMCs or CD56-depleted PBMCs were co-cultured with the cell tracker green stain (**B**) SK-N-MC, (**C**) SK-ES-1 EWS target cells at a ratio of 25:1 for 5 h. Co-cultures were stained with the LIVE/DEAD™ Fixable Yellow Dead Cell Stain and the percentage of dead target cells was assessed by flow cytometry. Presented as mean ± SEM, *n* = 5, a two-way ANOVA was performed, ** *p* < 0.01, *** *p* < 0.001 and **** *p* < 0.0001. (**D**,**E**) PBMCs were treated ± MG1 at 1 PFU/cell for 48 h. PBMCs were co-cultured at a ratio of 10:1 with EWS cell lines for 4 h. The percentage of degranulating NK cells was detected by staining with CD56, CD3 and CD107a antibodies and flow cytometry. (**D**) Representative gating strategy for SK-N-MC target cells, showing lymphocyte gating, CD56+CD3- NK cell gating and the % CD107a+ degranulating NK cells under conditions with PBMC with no target cells, untreated PBMC with SK-N-MC target cells and MG1-stimualted PBMC with SK-N-MC target cells. (**E**) Summary of results presented as fold change in degranulating NK cells relative to untreated PBMC control. Presented as mean ± SEM, *n* = 3, a two-way ANOVA was performed, * *p* < 0.05, ** *p* < 0.01. (**F**) PBMCs were treated ± MG1 at 1 PFU/cell for 48 h and then treated ±1 mM EGTA for 30 min. PBMCs were then co-cultured with SK-N-MC EWS target cells at a ratio of 25:1 for 5 h. Cells were stained with the LIVE/DEAD™ Fixable Yellow Dead Cell Stain and the percentage of dead target cells was assessed using flow cytometry. Presented as mean ± SEM, *n* = 4, a two-way ANOVA was performed, *** *p* < 0.001, **** *p* < 0.0001.

To investigate the importance of NK cells in this activity, we repeated these experiments using PBMCs from which the NK cells had been depleted using magnetic immunoselection. Flow cytometry confirmed successful depletion of the NK cells from PBMCs (~86% depleted, Figure 5A), which resulted in a significant reduction in killing of the four EWS cell lines (Figure 5B,C and Figure S5A,B). These results demonstrate that MG1 activates NK cell-mediated cytotoxicity towards EWS cell lines. Accordingly, NK cell degranulation in response to EWS cell lines (cultured as either monolayers or spheroids) was significantly enhanced by pre-treatment with MG1 (Figure 5D,E and Figure S5C), suggesting that perforin/granzyme-mediated cytotoxicity was responsible for EWS cell death. The delivery of granzymes to target cells is dependent on the calcium-mediated oligomerisation of perforin, and treatment of PBMCs with the calcium chelator EGTA inhibited unstimulated and MG1-stimulated PBMC-mediated killing of both SK-N-MC and TTC-466 EWS cell lines, supporting a role for the perforin-dependent granule-mediated killing of the EWS cells (Figure 5F and Figure S5D) [56].

## 4. Discussion

Oncolytic viruses offer an innovative approach for stimulating the immune response against hard-to-treat solid tumours such as EWS. In this study, we present a comprehensive study of the effect of MG1 against EWS, and demonstrate that the oncolytic rhabdovirus, Maraba virus MG1, is a candidate OV for the treatment of EWS. To highlight the potential clinical relevance of our study, we used complementary EWS model systems, including those recently derived from patient samples and cell lines that exhibit resistance to chemotherapy drugs used to treat EWS. Mechanistic studies show that MG1 can kill EWS cells directly and mediate the immune-based killing of EWS cells, specifically through the action of NK cells.

When comparing OVs, MG1 frequently out-performs other rhabdoviruses and OVs from other virus families in terms of direct oncolytic action in vitro, which is consistent with our findings in EWS [25,32,52]. While the precise mechanism of cell death induced by MG1 is yet to be elucidated, it exhibits similar properties to vesicular stomatitis virus, a closely related member of the rhabdovirus family, which is well established to induce apoptosis [57]. Previous publications have demonstrated that MG1 induces direct lysis of several solid cancer cell types including melanoma, breast, colon, ovarian and lung cancer cell lines [23,25,41,58,59,60]. However, the action of MG1 against EWS remains relatively unexplored. Le Bouef et al. demonstrated the direct oncolytic activity of MG1 against the long-established EWS cell line, RD-ES, in monolayer cultures. The same study showed that primary tumour tissue isolated from a variety of sarcoma types could support MG1 replication and that murine S180 sarcomas (of fibrosarcoma origin) were susceptible to MG1 in vivo [32]. However, these studies did not include EWS patient samples or EWS cell lines other than RD-ES. Our findings build substantially on this previous work, establishing the efficacy of MG1 in more complex 3D spheroid models, chemotherapy-resistant cell lines and PDES cell cultures and demonstrates that MG1 is capable of infecting, replicating and lysing EWS cells.

Studies of MG1 and ovarian cancer demonstrated that cell surface expression of LDLRs was required for MG1 entry and that LDLR expression was reduced when culturing cells as spheroids compared to conventional monolayers, delaying oncolysis [52]. By contrast, our data demonstrated that LDLR was expressed in all the EWS cell models tested, and all were susceptible to MG1 oncolysis, albeit to varying levels. However, transcriptome data demonstrated heterogeneity of LDLR gene expression at the single-cell level, with substantial populations of EWS tumour cells lacking detectable LDLR gene expression. The heterogeneity of LDLR expression could be viewed as a barrier to the use of MG1 in EWS, as large populations of tumour cells may not be amenable to infection. However, LDLR expression is dynamic and the *LDLR* gene, along with genes encoding components of cholesterol biosynthesis, are expressed in response to a drop in cellular cholesterol. This allows cells to endocytose cholesterol from the extracellular environment (via LDLRs) and to synthesise new cholesterol, restoring the pool [61]. The heterogeneity of *LDLR* gene expression observed in EWS scRNAseq might therefore reflect a snapshot of the differential requirements for cholesterol amongst tumour cells, for example, a greater requirement in those cells undergoing proliferation at the time of analysis.

Interestingly, *ERG2* has been identified as a direct target of EWSR1::ETS transcription factors, which drives EWS tumorigenicity [62]. The ERG2 transcription factor regulates the transcription of enzymes involved in mevalonate pathways, which results in increased cholesterol production. Moreover, Atorvastatin and Simvastatin, HMG-CoA reductase inhibitors, limit production of cholesterol, and have been shown to reduce EWS cell line proliferation in vitro and in vivo [62]. Pivotally, it has been long established that treatment with statins induces cell surface expression of LDLRs, and as such, future work should explore the potential synergy of statins with MG1 in EWS, to enhance viral entry and oncolysis [63].

Resistance to oncolysis affects just one mechanism of action of OVs. Building on models of OV action, we suggest that there will be oncolysis of some EWS cells and this will release damage-associated molecular patterns, pathogen-associated molecular patterns and tumour antigens [64]. This will occur within the context of an anti-viral response mediated by non-malignant cells, including the production of IFN-I and the activation of NK cells. We have previously shown that OVs can stimulate the production of IFN-I from monocytes, that the activation of NK cells by OVs is IFN-I dependent in vitro and that the peak of IFN-I responses coincides with the peak of NK cell activation in patients receiving an intravenous OV [27,28]. Furthermore, the OV treatment of PBMCs results in the expression of NK cell cytotoxic machinery, enhancing the ability of OV-activated NK cells to kill tumour cells. Therefore, populations of EWS cells that are not infected and lysed by MG1 directly could nevertheless find themselves in an environment in which NK cells are activated and capable of EWS recognition and killing. Whilst IFN-I production by healthy cells will impair EWS oncolysis, it is expected to promote NK cell activation and tumour lysis. Further, although not tested here, many studies have demonstrated that OVs also promote T cell activity against tumours, for example, MG1 monotherapy enhanced T cell responses against melanoma [23].

A recent study describes a combination of an oncolytic herpes simplex virus (rRp450) with trabectedin, a chemotherapeutic agent that depletes pro-tumour macrophage populations. Combined OV and trabectidin treatmentresulted in increased tumour regression across three xenograft models of EWS (A673, CHLA258 and EW5) when compared with either agent alone [35]. The synergistic effect of these agents has been associated with trabectedin’s ability to disrupt anti-viral responses in tumour cells and enhance immune activation, in part due to a reduction in M2-like macrophage populations [34,35]. Importantly, Maraba virus is a (−)ssRNA virus amenable to genetic manipulation and its activity can be modulated by the inclusion of transgenes. MG1 encoding the cancer/testis antigen MAGE-A3 has been used as part of an OV/tumour vaccine approach and MG1 encoding microRNA molecules have been used to skew tumour-infiltrating macrophages from an M2-like (pro-tumour) immunosuppressive phenotype to an M1-like pro-inflammatory/anti-tumour phenotype in ovarian cancer models [65]. Similar modifications or combination strategies may have the potential to enhance the activity of MG1 against EWS and overcome the immunosuppressive TME. Combining the action of OVs with other immunotherapeutic approaches, such as immune checkpoint inhibitors or chimeric antigen receptor (CAR)-T cells is gaining traction in the treatment of other cancer types and may be an important consideration in OV therapy for EWS [64].

In this work, we opted for wholly human EWS model systems. In vivo studies of EWS largely involves the use of xenograft mice, which are not appropriate for the study of OVs due to a lack of a functional human immune system, which is essential to understand their full therapeutic potential [66]. An alternative approach is the use of humanised CD34+ haematopoietic stem cell-reconstituted mice harbouring human EWS tumours [66,67]. While these models hold great promise in the future for EWS research, particularly in the field of immunotherapy, they currently come with substantial limitations and do not fully represent EWS in patients [66,68]. Upon further development of humanised mouse models, where the reconstitution of immune cells better reflects relative immune cell proportions seen in humans, they are likely to become a more useful tool for preclinical testing of MG1, and indeed other OVs.

To date, OVs have demonstrated a good safety record in clinical trials [69,70]. The restriction of OV activity by IFN-I greatly reduces the infection of healthy tissue. Not surprisingly, side effects associated with OVs frequently resemble the symptoms of acute viral infections and are usually mild and transient in nature. A wild type Maraba virus strain has been shown to cause neuropathology in mice, but studies of MG1-based OVs in cats and non-human primates both reported an absence of adverse effects requiring clinical intervention [71,72,73]. Furthermore, several clinical trials incorporating MG1 are underway (clinical trial identifiers; NCT02285816, NCT02879760, NCT03618953 and NCT03773744) and one preliminary trial report suggests that adverse effects are similar to those observed with other OVs [74]. Although these clinical trials investigating MG1-based therapies for adult solid tumours are restricted to patients over 18 years of age, a separate phase I study evaluating a herpes simplex virus-based OV in paediatric and young adult patients (aged 12 to 21), including patients with bone and soft tissue sarcomas, demonstrated safety profiles comparable to those seen in adult populations [75].

## 5. Conclusions

In summary, using a variety of EWS human in vitro models, we have shown that MG1 can infect, replicate and kill EWS cells directly and activate NK cell cytotoxicity against tumours. This dual activity suggests that MG1, and engineered derivatives, hold promise for the treatment of EWS in the future.

## Data Availability

Datasets for tumours and cell lines [46,47] and scRNAseq data from tumours [19] used in this work can be accessed on the database of Genotypes and Phenotypes (dbGaP; phs000768) and Gene Expression Omnibus (GEO; GSE243347), respectively.

## Supplementary Materials

The following supporting information can be downloaded at: https://www.mdpi.com/article/10.3390/cancers17203319/s1, Supplementary Materials and methods, Table S1. Cell lines and primary cell cultures, Table S2. Antibodies, fluorescent stains, other reagents and buffers, Figure S1. EWS cell line sensitivity to direct oncolysis by MG1 is superior to other molecularly distinct OVs, Figure S2. EWS cell line spheroid cultures, Figure S3. Characterisation of EWS cell cultures, Figure S4. IFNβ protects EWS cell lines against MG1 replication and Figure S5. MG1 stimulates immune-mediated killing of EWS targets by NK cell degranulation.

## Abbreviations

The following abbreviations are used in this manuscript:

DMEM	Dulbeccos modified eagles medium
EWS	Ewing sarcoma
NK	Natural killer
OV	Oncolytic virus
LDLR	Low-density lipoprotein receptor
PBMC	Peripheral blood mononuclear cells
PD-1	Programmed cell death protein-1
PD-L1	Programmed death ligand-1
CTLA-4	Cytotoxic T-lymphocyte associated protein-4
IFN	Interferon
HSV	Herpes simplex virus
IL	Interleukin
3D	3-Dimensional
TME	Tumour microenvironment
RNAseq	RNA sequencing
scRNAseq	Single-cell RNA sequencing
MSC	Mesenchymal stem cell

## References

[1] Ewing J. (1921). Diffuse Endothelioma of Bone. N. Y. Pathol. Soc..

[2] Prieur A., Tirode F., Cohen P., Delattre O. (2004). EWS/FLI-1 Silencing and Gene Profiling of Ewing Cells Reveal Downstream Oncogenic Pathways and a Crucial Role for Repression of Insulin-Like Growth Factor Binding Protein 3. Mol. Cell. Biol..

[3] Hancock J.D., Lessnick S.L. (2008). A transcriptional profiling meta-analysis reveals a core EWS-FLI gene expression signature. Cell Cycle.

[4] Guillon N., Tirode F., Boeva V., Zynovyev A., Barillot E., Delattre O. (2009). The oncogenic EWS-FLI1 protein binds in vivo GGAA microsatellite sequences with potential transcriptional activation function. PLoS ONE.

[5] Bailly R.A., Bosselut R., Zucman J., Cormier F., Delattre O., Roussel M., Thomas G., Ghysdael J. (1994). DNA-binding and transcriptional activation properties of the EWS-FLI-1 fusion protein resulting from the t(11;22) translocation in Ewing sarcoma. Mol. Cell. Biol..

[6] Sorensen P.H., Lessnick S.L., Lopez-Terrada D., Liu X.F., Triche T.J., Denny C.T. (1994). A second ewing’s sarcoma translocation, t(21;22), fuses the EWS gene to another ETS-family transcription factor, ERG. Nat. Genet..

[7] Delattre O., Zucman J., Plougastel B., Desmaze C., Melot T., Peter M., Kovar H., Joubert I., De Jong P., Rouleau G. (1992). Gene fusion with an ETS DNA-binding domain caused by chromosome translocation in human tumours. Nature.

[8] Brennan B., Kirton L., Marec-Berard P., Marec-Berard J., Gelderblom H., Gaspar N., Strauss S.J., Urgelles A.S., Anderton J., Laurence V. (2020). Comparison of two chemotherapy regimens in Ewing sarcoma (ES): Overall and subgroup results of the Euro Ewing 2012 randomized trial (EE2012). J. Clin. Oncol..

[9] Zöllner S.K., Amatruda J.F., Bauer S., Collaud S., de Álava E., DuBois S.G., Hardes J., Hartmann W., Kovar H., Metzler M. (2021). Ewing sarcoma—Diagnosis, treatment, clinical challenges and future perspectives. J. Clin. Med..

[10] Gaspar N., Hawkins D.S., Dirksen U., Lewis I.J., Ferrari S., Le Deley M.-C., Kovar H., Grimer R., Whelan J., Claude L. (2015). Ewing sarcoma: Current management and future approaches through collaboration. J. Clin. Oncol..

[11] Sun Q., Hong Z., Zhang C., Wang L., Han Z., Ma D. (2023). Immune checkpoint therapy for solid tumours: Clinical dilemmas and future trends. Signal Transduct. Target. Ther..

[12] Tawbi H.A., Burgess M., Bolejack V., Van Tine B.A., Schuetze S.M., Hu J., D’Angelo S., Attia S., Riedel R.F., Priebat D.A. (2017). Pembrolizumab in advanced soft-tissue sarcoma and bone sarcoma (SARC028): A multicentre, two-cohort, single-arm, open-label, phase 2 trial. Lancet.

[13] Thanindratarn P., Dean D.C., Nelson S.D., Hornicek F.J., Duan Z. (2019). Advances in immune checkpoint inhibitors for bone sarcoma therapy. J. Bone Oncol..

[14] Spurny C., Kailayangiri S., Jamitzky S., Altvater B., Wardelmann E., Dirksen U., Hardes J., Hartmann W., Rossig C. (2018). Programmed cell death ligand 1 (PD-L1) expression is not a predominant feature in Ewing sarcomas. Pediatr. Blood Cancer.

[15] Crompton B.D., Stewart C., Taylor-Weiner A., Alexe G., Kurek K.C., Calicchio M.L., Kiezun A., Carter S.L., Shukla S.A., Mehta S.S. (2014). The genomic landscape of pediatric Ewing sarcoma. Cancer Discov..

[16] Stahl D., Gentles A.J., Thiele R., Gütgemann I. (2019). Prognostic profiling of the immune cell microenvironment in Ewing’s Sarcoma Family of Tumors. Oncoimmunology.

[17] Kuo C., Giannikou K., Wang N., Warren M., Goodspeed A., Shillingford N., Hayashi M., Raredon M.S.B., Amatruda J.F. (2025). Tumor-associated stroma shapes the spatial tumor immune microenvironment of primary Ewing sarcomas. bioRxiv.

[18] Cillo A.R., Mukherjee E., Bailey N.G., Onkar S., Daley J., Salgado C., Li X., Liu D., Ranganathan S., Burgess M. (2022). Ewing Sarcoma and Osteosarcoma Have Distinct Immune Signatures and Intercellular Communication Networks. Clin. Cancer Res..

[19] Visser L.L., Bleijs M., Margaritis T., van de Wetering M., Holstege F.C.P., Clevers H. (2023). Ewing Sarcoma Single-cell Transcriptome Analysis Reveals Functionally Impaired Antigen-presenting Cells. Cancer Res. Commun..

[20] Berghuis D., Santos S.J., Baelde H.J., Taminiau A.H., Egeler R.M., Schilham M.W., Hogendoorn P.C., Lankester A.C. (2011). Pro-inflammatory chemokine-chemokine receptor interactions within the Ewing sarcoma microenvironment determine CD8+ T-lymphocyte infiltration and affect tumour progression. J. Pathol..

[21] De Angulo G., Hernandez M., Morales-Arias J., Herzog C.E., Anderson P., Wolff J., Kleinerman E.S. (2007). Early lymphocyte recovery as a prognostic indicator for high-risk Ewing sarcoma. J. Pediatr. Hematol. Oncol..

[22] Jhawar S.R., Thandoni A., Bommareddy P.K., Hassan S., Kohlhapp F.J., Goyal S., Schenkel J.M., Silk A.W., Zloza A. (2017). Oncolytic viruses-natural and genetically engineered cancer immunotherapies. Front. Oncol..

[23] Armstrong E., Chiu M.K.L., Foo S., Appleton L., Nenclares P., Patrikeev A., Mohan N., Mclaughlin M., Bozhanova G., Hoebart J. (2024). Combination of oncolytic Maraba virus with immune checkpoint blockade overcomes therapy resistance in an immunologically cold model of advanced melanoma with dysfunctional T-cell receptor signalling. J. Immunother. Cancer.

[24] Müller L., Berkeley R., Barr T., Ilett E., Errington-Mais F. (2020). Past, present and future of oncolytic reovirus. Cancers.

[25] Brun J., McManus D., Lefebvre C., Hu K., Falls T., Atkins H., Bell J.C., McCart J.A., Mahoney D., Stojdl D.F. (2010). Identification of genetically modified maraba virus as an oncolytic rhabdovirus. Mol. Ther..

[26] Aref S., Castleton A.Z., Bailey K., Burt R., Dey A., Leongamornlert D., Mitchell R.J., Okasha D., Fielding A.K. (2020). Type 1 Interferon Responses Underlie Tumor-Selective Replication of Oncolytic Measles Virus. Mol. Ther..

[27] Wantoch M., Wilson E.B., Droop A.P., Phillips S.L., Coffey M., El-Sherbiny Y.M., Holmes T.D., Melcher A.A., Wetherill L.F., Cook G.P. (2022). Oncolytic virus treatment differentially affects the CD56 dim and CD56 bright NK cell subsets in vivo and regulates a spectrum of human NK cell activity. Immunology.

[28] Parrish C., Scott G.B., Migneco G., Scott K., Steele L.P., Ilett E., West E.J., Hall K., Selby P.J., Buchanan D. (2015). Oncolytic reovirus enhances rituximab-mediated antibody-dependent cellular cytotoxicity against chronic lymphocytic leukaemia. Leukemia.

[29] Lacroix J., Kis Z., Josupeit R., Schlund F., Stroh-Dege A., Frank-Stöhr M., Leuchs B., Schlehofer J.R., Rommelaere J., Dinsart C. (2018). Preclinical testing of an oncolytic parvovirus in ewing sarcoma: Protoparvovirus H-1 induces apoptosis and lytic infection in vitro but fails to improve survival in vivo. Viruses.

[30] Abdelbary H., Brown C.W., Werier J., Bell J. (2014). Using Targeted Virotherapy to Treat a Resistant Ewing Sarcoma Model: From the Bedside to the Bench and Back. Sci. World J..

[31] Eshun F.K., Currier M.A., Gillespie R.A., Fitzpatrick J.L., Baird W.H., Cripe T.P. (2010). VEGF Blockade Decreases Tumor Uptake of Systemic Oncolytic Herpes Virus but Enhances Therapeutic Efficacy When Given After Virotherapy HHS Public Access Author manuscript. Gene Ther..

[32] Le Boeuf F., Selman M., Son H.H., Bergeron A., Chen A., Tsang J., Butterwick D., Arulanandam R., Forbes N.E., Tzelepis F. (2017). Oncolytic Maraba Virus MG1 as a Treatment for Sarcoma. Int. J. Cancer.

[33] Klose C., Berchtold S., Schmidt M., Beil J., Smirnow I., Venturelli S., Burkard M., Handgretinger R., Lauer U.M. (2019). Biological treatment of pediatric sarcomas by combined virotherapy and NK cell therapy. BMC Cancer.

[34] Denton N.L., Chen C.-Y., Hutzen B., Currier M.A., Scott T., Nartker B., Leddon J.L., Wang P.-Y., Srinivas R., Cassady K.A. (2018). Myelolytic Treatments Enhance Oncolytic Herpes Virotherapy in Models of Ewing Sarcoma by Modulating the Immune Microenvironment. Mol. Ther. Oncolytics.

[35] Ringwalt E.M., Currier M.A., Glaspell A.M., Chen C.-Y., Cannon M.V., Cam M., Gross A.C., Gust M., Wang P.-Y., Boon L. (2024). Trabectedin promotes oncolytic virus antitumor efficacy, viral gene expression, and immune effector function in models of bone sarcoma. Mol. Ther. Oncol..

[36] Schober S.J., Schoening C., Eck J., Middendorf C., Lutsch J., Knoch P., von Ofen A.J., Gassmann H., Thiede M., Hauer J. (2023). The Oncolytic Adenovirus XVir-N-31 Joins Forces with CDK4/6 Inhibition Augmenting Innate and Adaptive Antitumor Immunity in Ewing Sarcoma. Clin. Cancer Res..

[37] Holmes T.D., El-Sherbiny Y.M., Davison A., Clough S.L., Blair G.E., Cook G.P. (2011). A Human NK Cell Activation/Inhibition Threshold Allows Small Changes in the Target Cell Surface Phenotype To Dramatically Alter Susceptibility to NK Cells. J. Immunol..

[38] Pahl J.H., Ruslan S.E.N., Buddingh E.P., Santos S.J., Szuhai K., Serra M., Gelderblom H., Hogendoorn P.C., Egeler R.M., Schilham M.W. (2012). Anti-EGFR antibody cetuximab enhances the cytolytic activity of natural killer cells toward osteosarcoma. Clin. Cancer Res..

[39] Verhoeven D.H., de Hooge A.S., Mooiman E.C., Santos S.J., Dam M.M.T., Gelderblom H., Melief C.J., Hogendoorn P.C., Egeler R.M., van Tol M.J. (2008). NK cells recognize and lyse Ewing sarcoma cells through NKG2D and DNAM-1 receptor dependent pathways. Mol. Immunol..

[40] El-Sherbiny Y.M., Holmes T.D., Wetherill L.F., Black E.V.I., Wilson E.B., Phillips S.L., Scott G.B., Adair R.A., Dave R., Scott K.J. (2015). Controlled infection with a therapeutic virus defines the activation kinetics of human natural killer cells in vivo. Clin. Exp. Immunol..

[41] Bourgeois-Daigneault M.-C., St-Germain L.E., Roy D.G., Pelin A., Aitken A.S., Arulanandam R., Falls T., Garcia V., Diallo J.-S., Bell J.C. (2016). Combination of Paclitaxel and MG1 oncolytic virus as a successful strategy for breast cancer treatment. Breast Cancer Res..

[42] Hassanzadeh G., Naing T., Graber T., Jafarnejad S.M., Stojdl D.F., Alain T., Holcik M. (2019). Characterizing cellular responses during oncolytic maraba virus infection. Int. J. Mol. Sci..

[43] Wilson B.J., Owston H.E., Iqbal N., Giannoudis P.V., McGonagle D., Pandit H., Pampadykandathil L.P., Jones E., Ganguly P. (2024). In Vitro Osteogenesis Study of Shell Nacre Cement with Older and Young Donor Bone Marrow Mesenchymal Stem/Stromal Cells. Bioengineering.

[44] Roundhill E.A., Jabri S., Burchill S.A. (2019). ABCG1 and Pgp identify drug resistant, self-renewing osteosarcoma cells. Cancer Lett..

[45] Roundhill E.A., Chicon-Bosch M., Jeys L., Parry M., Rankin K.S., Droop A., Burchill S.A. (2021). RNA sequencing and functional studies of patient-derived cells reveal that neurexin-1 and regulators of this pathway are associated with poor outcomes in Ewing sarcoma. Cell. Oncol..

[46] Brohl A.S., Solomon D.A., Chang W., Wang J., Song Y., Sindiri S., Patidar R., Hurd L., Chen L., Shern J.F. (2014). The Genomic Landscape of the Ewing Sarcoma Family of Tumors Reveals Recurrent STAG2 Mutation. PLoS Genet..

[47] Brohl A.S., Sindiri S., Wei J.S., Milewski D., Chou H.-C., Song Y.K., Wen X., Kumar J., Reardon H.V., Mudunuri U.S. (2021). Immuno-transcriptomic profiling of extracranial pediatric solid malignancies. Cell Rep..

[48] Miyagawa Y., Okita H., Nakaijima H., Horiuchi Y., Sato B., Taguchi T., Toyoda M., Katagiri Y.U., Fujimoto J., Hata J.-I. (2008). Inducible Expression of Chimeric EWS/ETS Proteins Confers Ewing’s Family Tumor-Like Phenotypes to Human Mesenchymal Progenitor Cells. Mol. Cell. Biol..

[49] Rennerfeldt D.A., Raminhos J.S., Leff S.M., Manning P., van Vliet K.J. (2019). Emergent heterogeneity in putative mesenchymal stem cell colonies: Single-cell time lapsed analysis. PLoS ONE.

[50] Stahl M., Ranft A., Paulussen M., Bölling T., Vieth V., Bielack S., Görtitz I., Braun-Munzinger G., Hardes J., Jürgens H. (2011). Risk of Recurrence and Survival After Relapse in Patients With Ewing Sarcoma. Pediatr. Blood Cancer.

[51] Fong E.L.S., Lamhamedi-Cherradi S.-E., Burdett E., Ramamoorthy V., Lazar A.J., Kasper F.K., Farach-Carson M.C., Vishwamitra D., Demicco E.G., Menegaz B.A. (2013). Modeling Ewing sarcoma tumors in vitro with 3D scaffolds. Proc. Natl. Acad. Sci. USA.

[52] Tong J.G., Valdes Y.R., Barrett J.W., Bell J.C., Stojdl D., McFadden G., McCart J.A., E DiMattia G., Shepherd T.G. (2015). Evidence for differential viral oncolytic efficacy in an in vitro model of epithelial ovarian cancer metastasis. Mol. Ther.-Oncolytics.

[53] Roundhill E.A., Vasconcelos E.J., Westhead D.R., Grissenberger S., Distel M., Burchill S.A. (2023). Abstract 4683: Developing human Ewing sarcoma in vitro models to prioritise new treatments. Cancer Res..

[54] Pasello M., Manara M.C., Scotlandi K. (2018). CD99 at the crossroads of physiology and pathology. J. Cell Commun. Signal..

[55] Marelli G., Howells A., Lemoine N.R., Wang Y. (2018). Oncolytic viral therapy and the immune system: A double-edged sword against cancer. Front. Immunol..

[56] Lopez J.A., Susanto O., Jenkins M.R., Lukoyanova N., Sutton V.R., Law R.H.P., Johnston A., Bird C.H., Bird P.I., Whisstock J.C. (2013). Perforin forms transient pores on the target cell plasma membrane to facilitate rapid access of granzymes during killer cell attack. Blood.

[57] Li W., Turaga R.C., Li X., Sharma M., Enadi Z., Tompkins S.N.D., Hardy K.C., Mishra F., Tsao J., Liu Z.-R. (2019). Overexpression of Smac by an Armed Vesicular Stomatitis Virus Overcomes Tumor Resistance. Mol. Ther.-Oncolytics.

[58] Alkayyal A.A., Tai L.-H., Kennedy M.A., de Souza C.T., Zhang J., Lefebvre C., Sahi S., Ananth A.A., Mahmoud A.B., Makrigiannis A.P. (2017). NK-cell recruitment is necessary for eradication of peritoneal carcinomatosis with an IL12-expressing Maraba virus cellular vaccine. Cancer Immunol. Res..

[59] Bourgeois-Daigneault M.-C., Roy D.G., Aitken A.S., El Sayes N., Martin N.T., Varette O., Falls T., St-Germain L.E., Pelin A., Lichty B.D. (2018). Neoadjuvant oncolytic virotherapy before surgery sensitizes triple-negative breast cancer to immune checkpoint therapy. Sci. Transl. Med..

[60] Mahoney D.J., Lefebvre C., Allan K., Brun J., Sanaei C.A., Baird S., Pearce N., Grönberg S., Wilson B., Prakesh M. (2011). Virus-Tumor Interactome Screen Reveals ER Stress Response Can Reprogram Resistant Cancers for Oncolytic Virus-Triggered Caspase-2 Cell Death. Cancer Cell.

[61] Horton J.D., Goldstein J.L., Brown M.S. (2002). SREBPs: Activators of the complete program of cholesterol and fatty acid synthesis in the liver. J. Clin. Invest..

[62] Buchou C., Laud-Duval K., van der Ent W., Grossetête S., Zaidi S., Gentric G., Corbé M., Müller K., Del Nery E., Surdez D. (2022). Upregulation of the Mevalonate Pathway through EWSR1-FLI1/EGR2 Regulatory Axis Confers Ewing Cells Exquisite Sensitivity to Statins. Cancers.

[63] Mabuchi H., Haba T., Tatami R., Miyamoto S., Sakai Y., Wakasugi T., Watanabe A., Koizumi J., Takeda R. (1981). Effects of an Inhibitor of 3-Hydroxy-3-Methylglutaryl Coenzyme a Reductase on Serum Lipoproteins and Ubiquinone-10 Levels in Patients with Familial Hypercholesterolemia. N. Engl. J. Med..

[64] Lin D., Shen Y., Liang T. (2023). Oncolytic virotherapy: Basic principles, recent advances and future directions. Signal Transduct. Target. Ther..

[65] Jennings V.A., Rumbold-Hall R., Migneco G., Barr T., Reilly K., Ingram N., Hilare I.S., Heaton S., Alzamel N., Jackson D. (2024). Enhancing oncolytic virotherapy by extracellular vesicle mediated microRNA reprograming of the tumour microenvironment. Front. Immunol..

[66] Petrescu D.I., Yustein J.T., Dasgupta A. (2024). Preclinical models for the study of pediatric solid tumors: Focus on bone sarcomas. Front. Oncol..

[67] Luo W., Hoang H., Liao Y., Pan J., Ayello J., Cairo M.S. (2023). A humanized orthotopic mouse model for preclinical evaluation of immunotherapy in Ewing sarcoma. Front. Immunol..

[68] Tian H., Lyu Y., Yang Y.-G., Hu Z. (2020). Humanized Rodent Models for Cancer Research. Front. Oncol..

[69] Schirrmacher V. (2020). Cancer Vaccines and Oncolytic Viruses Exert Profoundly Lower Side Effects in Cancer Patients than Other Systemic Therapies: A Comparative Analysis. Biomedicines.

[70] Gao P., Ding G., Wang L. (2021). The efficacy and safety of oncolytic viruses in the treatment of intermediate to advanced solid tumors: A systematic review and meta-analysis. Transl. Cancer Res..

[71] Maia-Farias A., Lima C., Freitas P., Diniz D., Rodrigues A., Quaresma J., Diniz C.P., Diniz J. (2020). Early and late neuropathological features of meningoencephalitis associated with Maraba virus infection. Braz. J. Med. Biol. Res. Rev. Bras. Pesqui Med. Biol..

[72] Hummel J., Bienzle D., Morrison A., Cieplak M., Stephenson K., DeLay J., Woods J.P., Lichty B.D., Bridle B.W. (2017). Maraba virus-vectored cancer vaccines represent a safe and novel therapeutic option for cats. Sci. Rep..

[73] Pol J.G., Acuna S.A., Yadollahi B., Tang N., Stephenson K.B., Atherton M.J., Hanwell D., El-Warrak A., Goldstein A., Moloo B. (2019). Preclinical evaluation of a MAGE-A3 vaccination utilizing the oncolytic Maraba virus currently in first-in-human trials. Oncoimmunology.

[74] Jonker D.J., Hotte S.J., Razak A.R.A., Renouf D.J., Lichty B., Bell J.C., Powers J., Breitbach C.J., Stojdl D.F., Stephenson K.B. (2017). Phase I study of oncolytic virus (OV) MG1 maraba/MAGE-A3 (MG1MA3), with and without transgenic MAGE-A3 adenovirus vaccine (AdMA3) in incurable advanced/metastatic MAGE-A3-expressing solid tumours: CCTG IND.214. J. Clin. Oncol..

[75] Moreno L., Teira P., Croop J.M., Gerber N.U., André N., Aerts I., Subias L.G., De Wilde B., Bautista F., Turpin B. (2023). A phase 1, first-in-child, multicenter study to evaluate the safety and efficacy of the oncolytic herpes virus talimogene laherparepvec in pediatric patients with advanced solid tumors. Front. Pediatr..

