# Supplementary material for: Oncolytic Maraba Virus MG1 Mediates Direct and Natural Killer Cell-Dependent Lysis of Ewing Sarcoma
Source: Cancers (Basel). 2025 Oct 14;17(20):3319. doi: 10.3390/cancers17203319 (PMC12562827; doi:10.3390/cancers17203319)
Supplement: Supplementary file 1 [file cancers-17-03319-s001.zip › cancers-3883614-supplementary.pdf]

Supplemental Material

Materials & Methods

Oncolytic viruses

Reovirus type 3 dearing strain (Reolysin) stocks were provided by Oncolytics Biotech, and virus was titred by plaque assay on L929 cells. HSV1716 was obtained from Virttu Biologics and propagated on Vero cell line, virus stocks were titred by plaque assay on Vero cell line. Coxsackievirus A21 strain Kuykendall (CVA21) was obtained from the ATCC, the virus was propagated on Mel624 cell line and titred using plaque assay on Mel624 cells. All virus stocks were stored at -80°C until use.

Table S1. Cell lines and primary cell cultures

Cell type	Origin	Details	Growth medium
**Cell lines**			
SK-N-MC	Human Ewing sarcoma	EWSR1::FLI1 (type 1) fusion; Female; age 14 years	DMEM:F12 + 10% FBS
SK-ES-1	Human Ewing sarcoma	EWSR1::FLI1 (type 2) fusion; Male; age 18 years	McCoys 5A + 10% FBS
TC-32	Human Ewing sarcoma	EWSR1::FLI1 (type 1) fusion; Female; age 17 years	RPMI + 10 % FBS
TTC-466	Human Ewing sarcoma	EWSR1::ERG fusion; Female; 4 years	RPMI + 10 % FBS
Vero	Kidney epithelial cells (African green monkey)	N/A	DMEM + 10 % FBS
Mel624	Melanoma	N/A	DMEM + 10 % FBS
L929	Normal subcutaneous areolar and adipose tissue of a 100-day-old male C3H/An mouse	N/A	DMEM + 10 % FBS

**Primary cell cultures**			
MSC	Human Bone marrow-derived mesenchymal stem cells	Male; age 22 years	StemMACS™ MSC Expansion Media +100 units of penicillin and 0.1 mg/mL streptomycin
CCRG1-L-017	Human Ewing sarcoma	EWSR1::FLI1 (type 1) fusion; Male; age 15 years	RPMI + 10% FBS + 100 units of penicillin and 0.1 mg/mL streptomycin
CCRG1-L-023	Human Ewing sarcoma	EWSR1::FLI1 (type 2); Male; age 11 years	RPMI + 10% FBS + 100 units of penicillin and 0.1 mg/mL streptomycin
CCRG1-L-066	Human Ewing sarcoma	EWSR1::ERG; Female; age 17 years	RPMI + 10% FBS + 100 units of penicillin and 0.1 mg/mL streptomycin
Peripheral blood mononuclear cells	Human Healthy donor	Adult healthy donor blood from apheresis cones	RPMI + 10% FBS

RPMI; Roswell Park Memorial Institute, DMEM; Dulbecco's Modified Eagles Medium

Table S2. **Antibodies, fluorescent stains, other reagents and buffers**

<table>
<tr><th colspan="5">Flow cytometry antibodies</th></tr>
<tr><th>Target protein</th><th>Target species</th><th>Isotype</th><th>Fluorophore</th><th>Manufacturer</th></tr>
<tr><td>CD3</td><td>Human</td><td>Mouse IgG2aκ</td><td>PerCP</td><td>Miltenyi Biotec; Bergisch Gladbach, Germany</td></tr>
<tr><td>CD56</td><td>Human</td><td>Mouse IgG1k</td><td>eFluor450</td><td>Thermofisher Scientific Inc; Waltham, MA, USA</td></tr>
<tr><td>CD107a</td><td>Human</td><td>Mouse IgG1k</td><td>PE</td><td>Biolegend; San Diego, CA, USA</td></tr>
<tr><td>CD99</td><td>Human</td><td>Mouse IgG2ak</td><td>PE</td><td>Biolegend; San Diego, CA, USA</td></tr>
<tr><td>LDLR</td><td>Human</td><td>Mouse IgG1</td><td>PE</td><td>R&D systems; Minneapolis, MN, USA</td></tr>
<tr><td>CD69</td><td>Human</td><td>Mouse IgG1k</td><td>PE</td><td>Biolegend; San Diego, CA, USA</td></tr>
<tr><td>CD317</td><td>Human</td><td>Recombinant human IgG1</td><td>PE</td><td>Miltenyi Biotec; Bergisch Gladbach, Germany</td></tr>
<tr><th colspan="5">ELISA antibodies</th></tr>
<tr><th colspan="3">Product</th><th colspan="2">Manufacturer</th></tr>
<tr><td colspan="3">Anti-human IFN-α mAb (MT1/3/5), unconjugated</td><td colspan="2">Mabtech; Stockholm, Sweeden</td></tr>
<tr><td colspan="3">Anti-human IFN-α mAb (MT2/4/6), biotin</td><td colspan="2">Mabtech; Stockholm, Sweeden</td></tr>
<tr><td colspan="3">Human IFN-beta ELISA Kit - Quantikine</td><td colspan="2">R&D systems; Minneapolis, MN, USA</td></tr>
<tr><th colspan="5">Fluorescent stains</th></tr>
</table>

Product	Manufacturer
CellTracker™ Green CMFDA Dye	Thermofisher Scientific Inc; Waltham, MA, USA
LIVE/DEAD® Fixable Yellow Dead Cell Stain Kit	Thermofisher Scientific Inc; Waltham, MA, USA
**Other reagents**	
**Product**	**Manufacturer**
CellTiter-Glo® reagent	Promega; Madison, WI, USA
Brefeldin A	Biolegend; San Diego, CA, USA
Lymphoprep	StemCell Technologies
Foetal Bovine Serum	Sigma-Aldrich; Saint Louis, MO, USA
Methylthialazole Tetrazolium; MTT	Sigma-Aldrich; Saint Louis, MO, USA
CD56 Microbeads Human	Miltenyi Biotec; Bergisch Gladbach, Germany
Accutase	Thermofisher Scientific Inc; Waltham, MA, USA
EDTA	Sigma-Aldrich; Saint Louis, MO, USA
Human IFNβ recombinant protein	PeproTech; Rocky Hill, NJ, USA
**Buffers**	
MACS buffer	PBS + 1% FBS + 0.4% 0.5M EDTA
FACS buffer	PBS + 10% FBS + 0.1% sodium azide

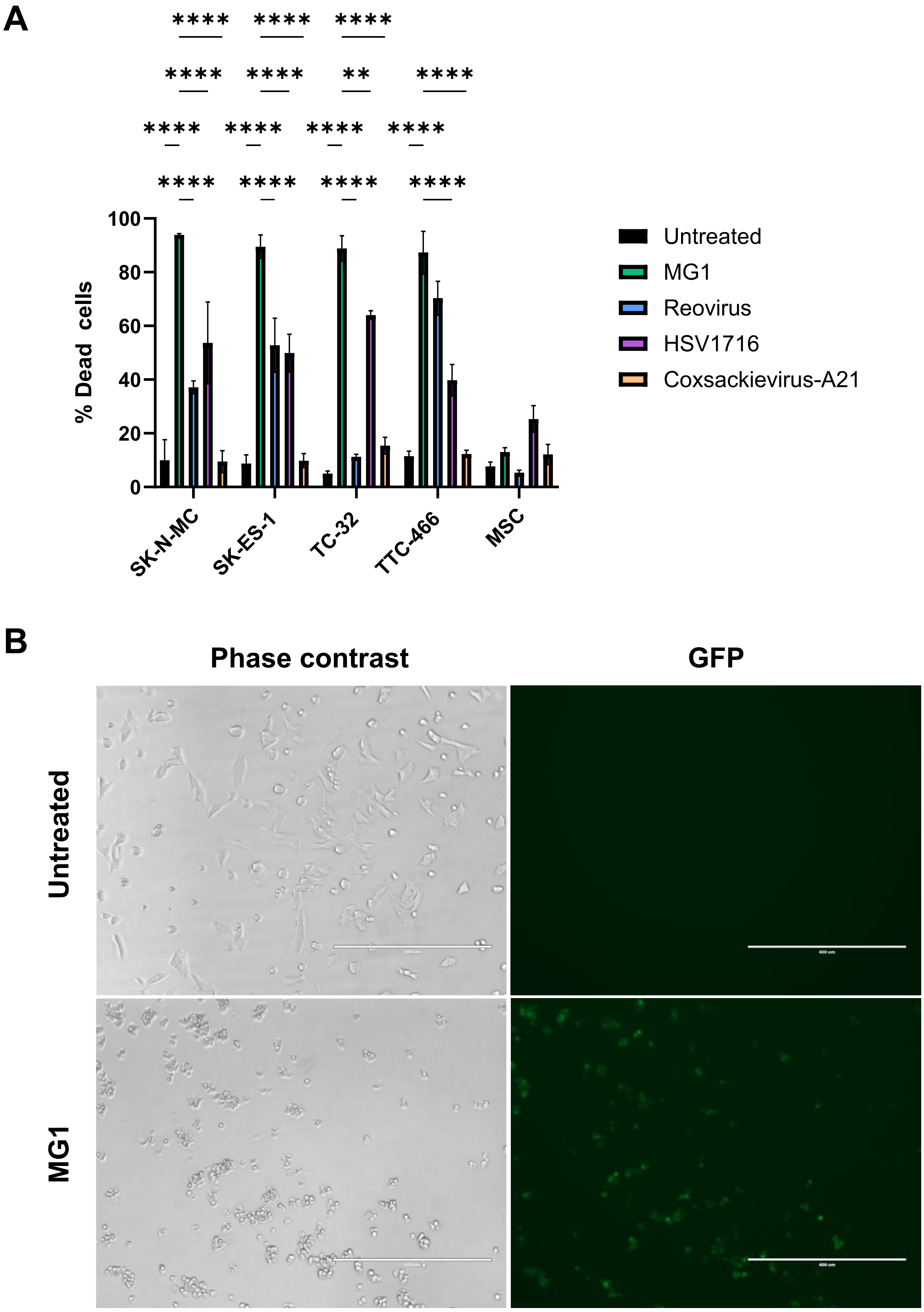
A
****
****
****
**
% Dead cells
100
80
60
40
20
0
SK-N-MC
SK-ES-1
TC-32
TTC-466
MSC
Untreated
MG1
Reovirus
HSV1716
Coxsackievirus-A21
B
Phase contrast
GFP
Untreated
MG1

Figure S1. EWS cell line sensitivity to direct oncolysis by MG1 is superior to other molecularly distinct OVs. (**A**) Four EWS cell lines (SK-N-MC, SK-ES-1, TC-32 and TTC-466) and MSCs were treated ± MG1, Reovirus, HSV1716 or Coxsackievirus-A21 at 1 PFU/cell. After 48 h, cells were stained with LIVE/DEAD™ Fixable Yellow Dead Cell Stain and analysed using flow cytometry, the graph shows mean ± SEM, n = 3, a two-way ANOVA was performed to compare % dead cells following MG1treatment to untreated control and other OVs *$^{*}p<0.05$, $^{****}p<0.0001$*. (**B**) TC-32 cell lines were treated ± MG1-GFP at 1 PFU/cell. After 24 h cells were imaged using EVOS fluorescent microscope and phase contrast and GFP images taken at 10x magnification. Scale bar = 400 μm.

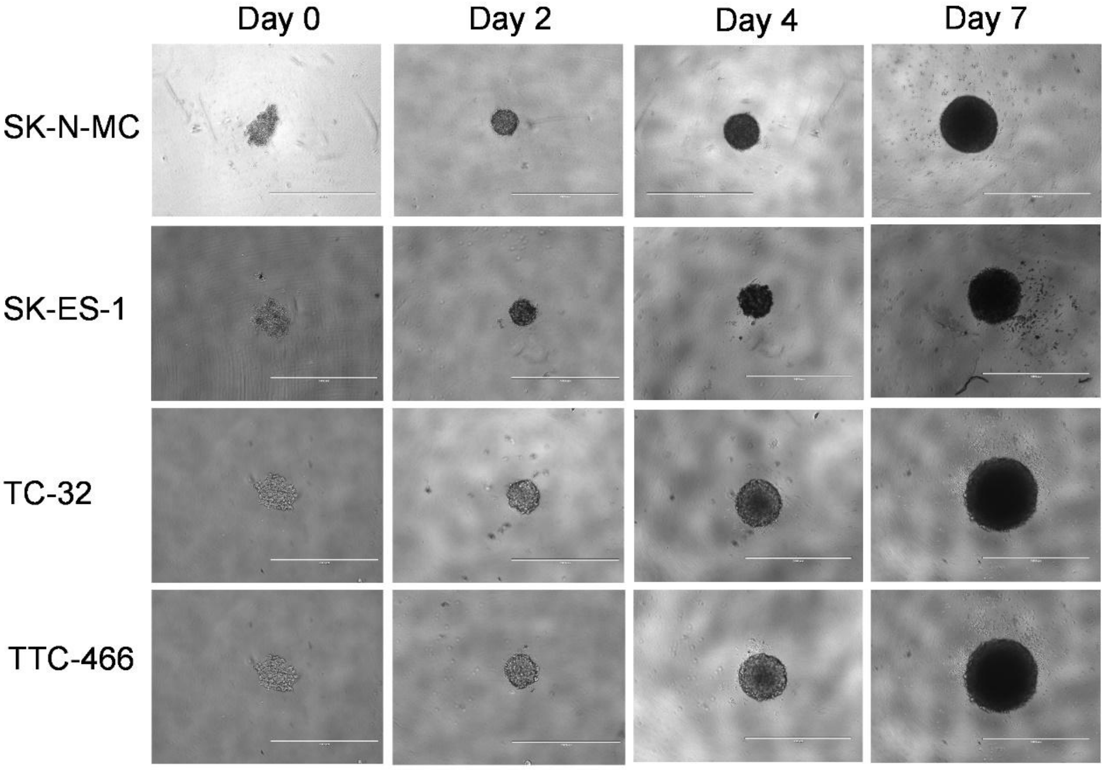

Figure S2. EWS cell line spheroid cultures. EWS cell lines were seeded into low adhesion 96 well plates at 2000 cells/ well for 7 days. Spheroid formation was tracked over 7 days, where spheroids were imaged using EVOS microscope at 4x magnification on days 0, 2, 4 and 7. Scale bar = 1 mm.

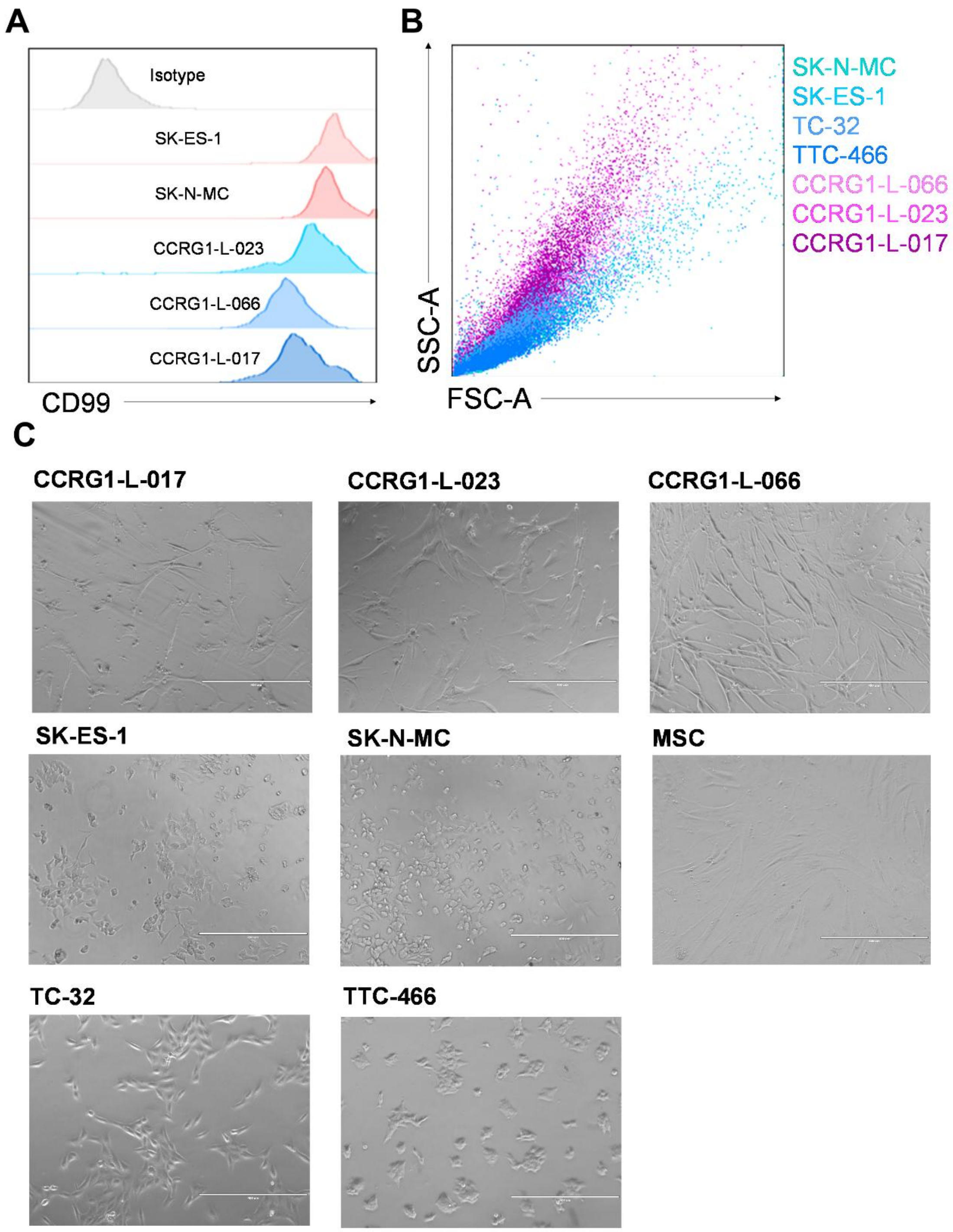

Figure S3. Characterisation of EWS cell cultures. (**A**) EWS cell lines (SK-ES-1 and SK-N-MC) and PDES cell cultures (CCRG-L-023, CCRG-L-066, CCRG-L-017) were stained with a fluorescently conjugated anti-CD99 antibody or a matched isotype control, and CD99 expression assessed using flow cytometry. (**B**) PDES cultures (pink) and EWS cell lines (blue) FCS-SSC profiles assessed using flow cytometry, and overlayed using FlowJo V10.8.1 software. (**C**) PDES cell cultures, EWS cell lines and MSC were seeded into 12 well plates at 1 x 10^5 cells/well for 24 h. Cell cultures were imaged at 10x magnification. Scale bar = 400 μm.

A

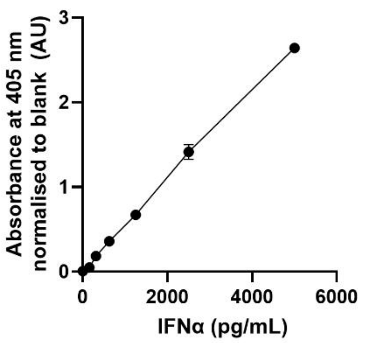

Cell type	Absorbance at 405 nm normalised to blank (AU)	IFNα (pg/mL)
SK-N-MC	0	ND
SK-ES-1	0	ND
TC-32	0	ND
TTC-466	0	ND
CCRG1-L-066	0	ND
CCRG1-L-023	0	ND
CCRG1-L-017	0	ND
MSC	0	ND

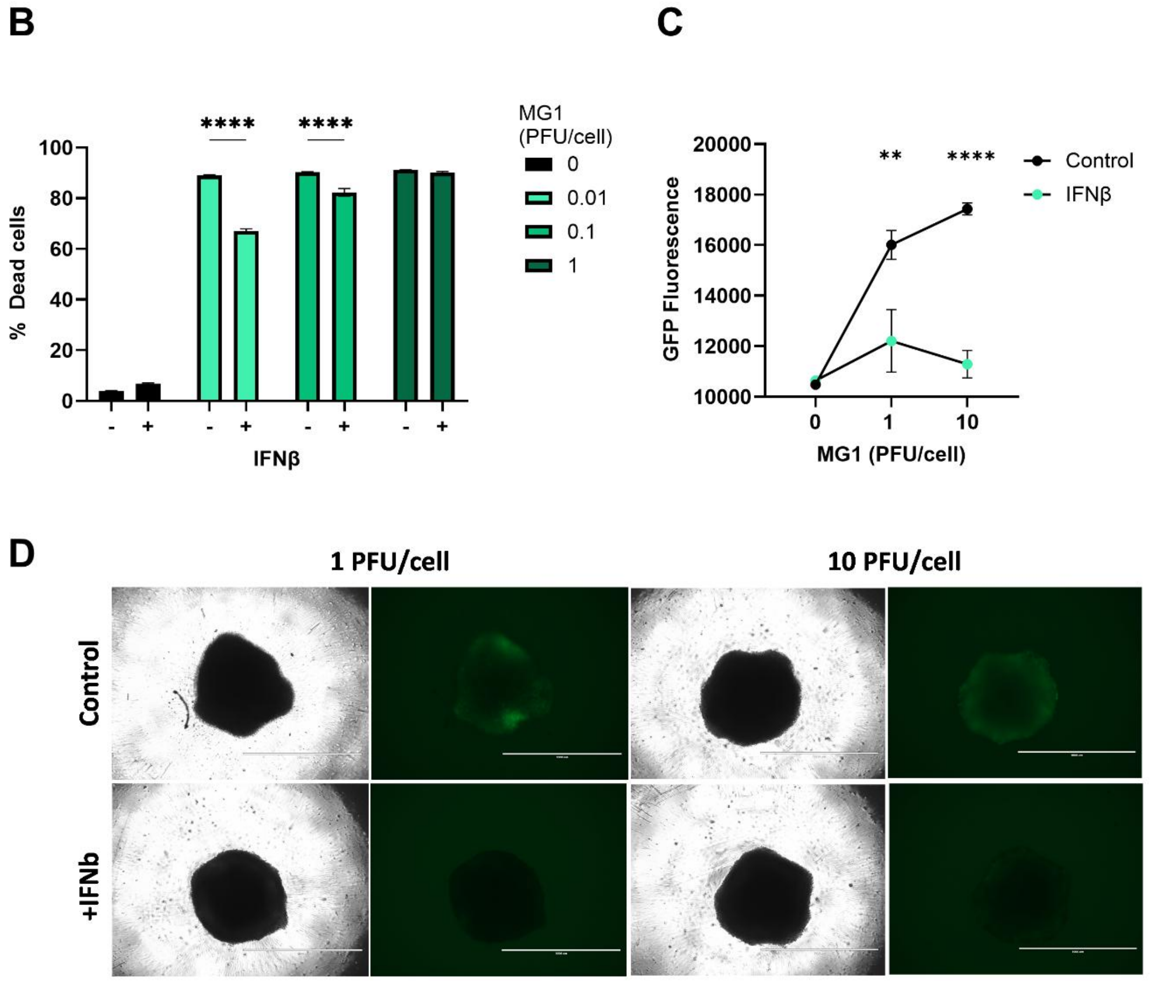

Figure S4. IFNβ protects EWS cell lines against MG1 replication. (**A**) PDES cell cultures, established cell lines and MSC were treated ± MG1 at 1 PFU/cell and cell-free supernatants were collected after 48 hours and screened alongside recombinant standards for IFNα using ELISA. Results show standard curve for ELISA standard and outputs for cell supernatants, ND = not detected. Presented as mean ± SEM, n=3. (**B**) SK-N-MC cells were treated ± IFNβ at 400 pg/mL for 24 hours, and then treated ± MG1 at 0.01, 0.1 and 1 PFU/cell for 48 hours. Cells were stained with LIVE/DEAD™ Fixable Yellow Dead Cell Stain and analysed using flow cytometry. Presented as mean ± SEM, n = 3, a two-way ANOVA was performed, ****$p<0.0001$. (**C,D**) SK-N-MC cells were seeded into low adhesion 96 well plates at 2000 cells/well to generate spheroids over 7 days. Spheroids were treated ± IFNβ at 400 pg/mL for 24 h and then treated

± MG1-GFP at 1 or 10 PFU/cell. After 24 h (**C**) GFP fluorescence was quantified using Cytation 5 plate reader at wavelength 485 nm, presented as mean ± SEM, $n = 3$, a two-way ANOVA was performed, ** *p<0.01, ****p<0.0001*. (**D**) cells were imaged using EVOS fluorescent microscope to obtain phase contrast and GFP images taken at 4x magnification. Scale bar = 1mm. Results presented are representative of a minimum of $n = 3$ independent experiments.

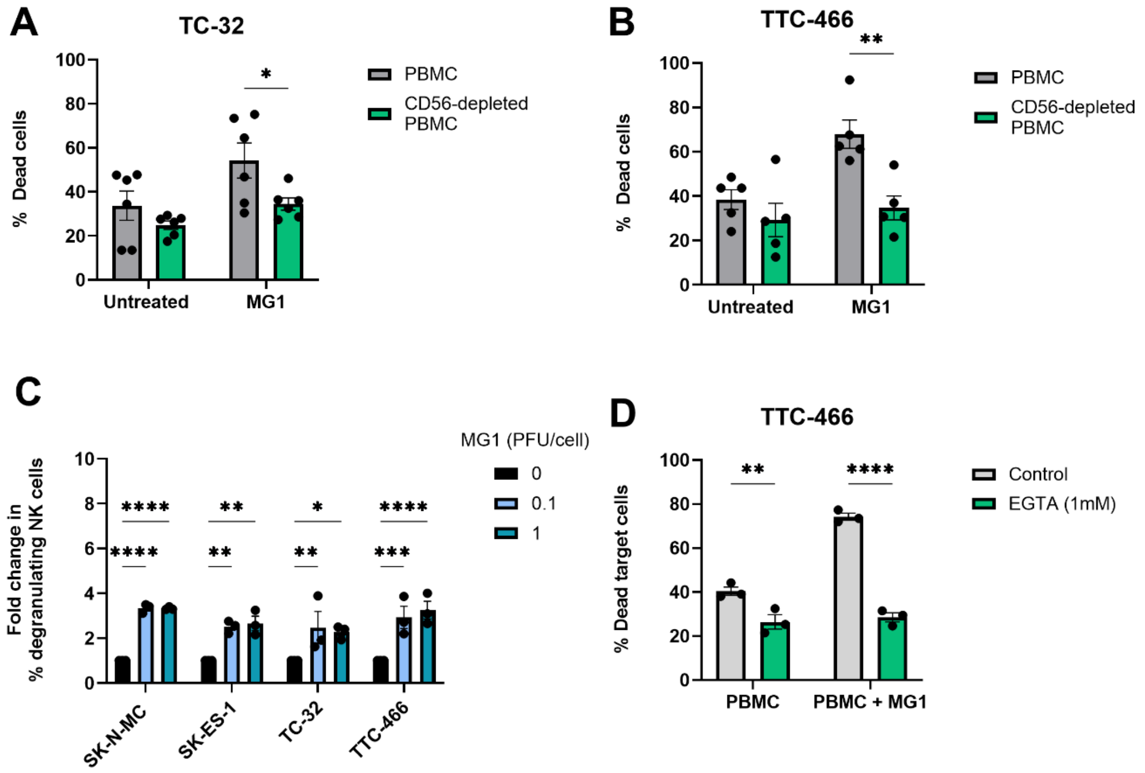

Figure S5. MG1 stimulates immune-mediated killing of EWS targets by NK cell degranulation. (**A,B**) NK cells were depleted from PBMC using CD56 magnetic bead selection and treated ± MG1 for 48 h. Whole PBMC or CD56-depleted PBMC were co-cultured with cell tracker green stained (**A**) TC-32 and (**B**) TTC-466 EWS target cells at a ratio of 25:1 for 5 h. Co-cultures were stained with LIVE/DEAD™ Fixable Yellow Dead Cell Stain and the percentage of dead target cells assessed by flow cytometry. Presented as mean ± SEM, $n \geq 5$, a two-way ANOVA was performed, * *p<0.05, **p<0.01*. (**C**) EWS cell lines spheroid cultures were generated over 7 days and PBMC were treated ± MG1 at 0.1 or 1 PFU/cell for 48 h. PBMC were then co-cultured at a ratio of 10:1 with EWS spheroids for 4 h. The percentage of degranulating NK cells was detected by staining with CD56, CD3 and CD107a antibodies and flow cytometry. Results presented as fold change in degranulating NK cells relative to untreated control. Presented as mean ± SEM, $n = 3$, a two-way ANOVA was performed, * *p<0.05, **p<0.01, ***p<0.001, ****p<0.0001*. (**D**) PBMC were treated ± MG1 at 1 PFU/cell for 48 h and then treated ± 1 mM EGTA for 30 minutes. PBMC were then co-cultured with TTC-466 EWS target cells at a ratio of 25:1 for 5 h. Cells were stained with LIVE/DEAD™ Fixable Yellow Dead Cell Stain and the percentage dead target cells assessed using flow cytometry. Presented as mean ± SEM, $n = 3$, a two-way ANOVA was performed, ** *p<0.051, ****p<0.0001*.
